# Exercise Intolerance in Young Physically Active Smokers Compared to Non‐Smokers During Sinusoidal Cycling

**DOI:** 10.1002/ejsc.70238

**Published:** 2026-08-24

**Authors:** Marta Borrelli, Asia Motalli, Nicholas Toninelli, Stefano Longo, Giuseppe Coratella, Emiliano Cè, Eloisa Limonta, Susanna Rampichini, Fabio Esposito

**Affiliations:** ^1^ Department of Biomedical Sciences for Health Università degli Studi di Milano Milan Italy

**Keywords:** cigarette, endurance, oxygen uptake kinetics, pulmonary oxygen uptake, time delay

## Abstract

The impact of cigarette smoking on the cardiorespiratory response to exhaustive exercise remains unclear. This study investigated the smoking effect on exercise tolerance by analyzing the cardiorespiratory and metabolic variables during an exhausting sinusoidal exercise in young physically active smokers (SM) without known cardiovascular and respiratory disease. Eight physically active male SM (22.1 ± 2.2 years; 79.9 ± 4.4 kg; maximum oxygen uptake, ${\dot{V}}_{O_{2}\;\max}$ 3385 ± 341 mL·min^−1^) and 8 non‐smokers (controls, 22.6 ± 1.4 years; 74.3 ± 7.9 kg; ${\dot{V}}_{O_{2}\;\max}$ 3623 ± 302 mL·min^−1^) performed an exhausting sinusoidal cycling exercise. The work rate varied sinusoidally around a midpoint 50 W below the lactate threshold (LT), with an amplitude of ± 50 W and a 4‐min cycle period. Midpoint, amplitude, and time‐delay between mechanical and metabolic signals of pulmonary ventilation (${\dot{V}}_{E}$), ${\dot{V}}_{O_{2}}$, and heart rate (HR) were assessed sinusoid‐by‐sinusoid, as well as blood lactate ([La^−^]) and rate of perceived exertion (RPE). Despite similar LT (199 ± 26 vs. 219 ± 28 W for SM and controls, respectively), SM exhibited a lower time to exhaustion (2461 ± 386 vs. 4512 ± 1359 s for SM and controls, respectively; *p* = 0.001) than controls. No differences between groups emerged in midpoint, amplitude, and time delays, as well as in [La^−^] and RPE. In SM, ${\dot{V}}_{O_{2}}$ midpoint remained constant, whereas in controls during the last cycle was higher compared to the first one (*p* = 0.001). In both groups, HR (*p* < 0.01) and ${\dot{V}}_{E}$ midpoint increased from the first to the last cycle (*p* < 0.05). The cigarette smoking‐induced exercise intolerance suggests metabolic and ventilatory impairments during sinusoidal cycle exercise even in young, physically active SM with no cardiovascular and respiratory disease.

## Highlights

- Cigarette smoking appears to negatively influence exercise tolerance in young, physically active males with no known lung or cardiovascular conditions.
- Notably, cardiorespiratory and metabolic response (i.e., midpoint, amplitude and time delays) to sinusoidal exercise was similar between groups, even though, smokers did not exhibit an increase in ${\dot{V}}_{O_{2}}$ and ${\dot{V}}_{E}$ midpoint throughout the exhaustion exercise.
- Overall, cigarette smoking‐induced exercise intolerance suggests metabolic and ventilatory impairments during sinusoidal cycle exercise, even in young physically active smokers.

## 1 Introduction

Cigarette smoking is the primary preventable cause of cardiovascular disease and mortality worldwide (World Health Organization 2017). The cigarette smoking‐compound harmful effects extend to the cardiorespiratory and muscular systems both at rest and during exercise (de Tarso Muller et al. 2019). In fact, carbon monoxide (CO) reduces the O_2_ delivery by binding to hemoglobin (HbCO) (de Tarso Muller et al. 2019; McDonough and Moffatt 1999) and nicotine over‐activates the sympathetic nervous system, leading to increased resting heart rate (HR) and peripheral vasoconstriction (Kobayashi et al. 2004; Papathanasiou et al. 2007). Additionally, tar from tobacco combustion elevates the pulmonary airway resistance and the work of breathing (de Tarso Muller et al. 2019; McDonough and Moffatt 1999). Furthermore, reactive oxygen species and other oxidants contribute to inflammation and impair skeletal muscle fibers function, specifically at the mitochondrial level (Degens et al. 2015).

The cigarette smoking‐related detrimental effects also involve the cardiorespiratory and metabolic response during exercise, contributing to exercise intolerance (de Tarso Muller et al. 2019). A lower pulmonary oxygen uptake (${\dot{V}}_{O_{2}}$) and pulmonary ventilation (${\dot{V}}_{E}$) at peak incremental exercise were observed not only in sedentary middle‐aged smokers (SM) with (Elbehairy et al. 2017) and without chronic obstructive pulmonary disease (Elbehairy et al. 2016; Sadaka et al. 2021) but also in young, physically active SM without known lung or cardiovascular problems (Borrelli et al. 2025; Papathanasiou et al. 2007). Additionally, cigarette smoking caused slower cardiorespiratory and metabolic kinetics during the recovery phase of maximal exercise (i.e., higher time constant, *τ*) attributable to a larger O_2_ debt (Borrelli et al. 2025). Even during a submaximal exercise, young SM exhibited a reduced exercise capacity at the same work rate, as evidenced by higher HR (Papathanasiou et al. 2007) and blood lactate concentration ([La^−^]) (Kobayashi et al. 2004), and by impaired gas exchange (i.e., higher ventilatory equivalent for oxygen, ${\dot{V}}_{E}/{\dot{V}}_{O_{2}}$ and for carbon dioxide, ${\dot{V}}_{E}/{\dot{V}}_{{\text{CO}}_{2}}$) (Kobayashi et al. 2004).

Although an increased neuromuscular fatigue associated with cigarette smoking was reported (de Tarso Muller et al. 2019; Degens et al. 2015), its impact on the cardiorespiratory and metabolic response to exhaustive exercise remains unclear. Although nicotine administration during moderate‐intensity exercise was shown to delay fatigue and increase the time to exhaustion (Mündel and Jones 2006), other circulating cigarette smoking constituents reduced exercise tolerance by augmenting muscle proteolysis, inhibiting protein synthesis and reducing O_2_ delivery (Degens et al. 2015). However, no studies examined so far cardiorespiratory and metabolic kinetics during exhaustive protocols. Indeed, assessing these kinetics is critical, as the dynamic ${\dot{V}}_{O_{2}}$ response immediately following exercise onset may influence the extent of muscle metabolic perturbation and, consequently, exercise tolerance (Jones and Burnley 2009).

To investigate the cardiorespiratory and metabolic kinetics over time, sinusoidal work rate protocols have been previously utilized (Borrelli et al. 2024; Fukuoka et al. 1995; Haouzi et al. 1993; Wigertz 1970). Despite being a convenient experimental model for laboratory studies, square‐wave and ramp exercise, which impose abrupt or continuously increasing workloads, are far away from actual exercise as performed in real life, wherein continuous oscillatory changes in workload occur. Conversely, the sinusoidal protocol provides a continuous yet smoothly varying workload, enabling deeper estimation of the phase shift of the cardiorespiratory and metabolic variables (Borrelli et al. 2024; Whipp and Ward 1990; Wigertz 1970). Similarly to an intermittent test, sinusoidal exercise permits dissociation between external power output and the internal metabolic stress (i.e., the extent of intramuscular metabolic perturbations), offering a unique opportunity to examine how rapidly and effectively the cardiorespiratory system adjusts to repeated relatively smooth transitions in work rate (Davies et al. 2017). By adjusting the frequency of the workload oscillations, the intramuscular metabolic perturbation can be modulated independently of the mean external power. Specifically, higher‐frequency oscillations in work rate were associated with a greater contribution of substrate‐level phosphorylation to muscular ATP resynthesis, particularly through increased phosphocreatine (PCr) breakdown (Davies et al. 2017; Girardi et al. 2024). Such information is particularly relevant in SM, in whom subtle impairments in dynamic physiological regulation may not be detectable during conventional incremental protocols.

Therefore, the objective of this study was to evaluate the cigarette smoking effect on exercise tolerance by monitoring the cardiorespiratory and metabolic variables during an exhausting sinusoidal task of moderate intensity (below the blood lactate accumulation threshold, LT) in young physically active SM without known lung or cardiovascular disease. Based on the findings of our previous study (Borrelli et al. 2024), we hypothesized that fatigue would not result in differences in time delay, which is the time lag between the mechanical and the cardiorespiratory and metabolic sines, as first cycles are expected to occur below the LT. However, because the protocol would be performed until exhaustion, lactate accumulation could occur in the final part of the test. However, we further hypothesized that SM would have longer time delays, and, consequently, reduced amplitude of the cardiorespiratory response and a shorter time to exhaustion compared to the control group, due to the above‐mentioned mechanisms induced by cigarette smoking.

## 2 Materials and Methods

### 2.1 Sample Size and Participants

Post‐hoc sensitivity analyses were performed to quantify the minimum detectable effect sizes given the achieved sample size. For the mixed‐model repeated‐measures ANOVA (within–between interaction) applied to the sinusoidal kinetic parameters (midpoint, amplitude, time delay), a sensitivity analysis (G*Power 3.1 Dusseldorf, Germany; test family: *F* tests; statistical test: ANOVA—repeated measures, within–between interaction) was conducted using *α* = 0.05, power = 0.80, 2 groups, 6 measurements, correlation among repeated measures = 0.50, and *ε* = 1. Under these conditions, the minimum detectable interaction effect size was Cohen's *f* = 0.269, indicating that the study was adequately powered to detect medium or larger effects. For the comparison of time‐to‐exhaustion (TTE), a separate sensitivity analysis was performed using a two‐sample *t*‐test (*α* = 0.05, power = 0.80, *n* = 8 per group). The minimum detectable effect size in this case was Cohen's *d* = 1.51. The observed effect size for TTE in the present data (*d* = 2.05) exceeded this threshold, confirming that this specific comparison was sufficiently powered to detect the between‐group difference. Therefore, 8 young physically active male SM (age: 22.1 ± 2.2 years; body mass: 79.9 ± 4.4 kg; stature: 1.79 ± 0.06 m; cigarettes per day: 11 ± 5; history of smoking: 6 ± 2 years) and 8 non‐smokers (controls; age: 22.6 ± 1.4 years; body mass: 74.3 ± 7.9 kg; stature: 1.79 ± 0.10 m), matched for age and exercise habits (International Physical Activity Questionnaire, IPAQ, 3846 ± 1850 vs. 3823 ± 1678 METs min^−1^·week^−1^, SM and controls respectively; *p* = 0.979) completed the protocol. The inclusion criteria for cigarette smoking were smoking at least 6 cigarettes per day for a minimum of two continuous years (Okuyemi et al. 2002). The exclusion criteria for both groups were (i) cardiorespiratory and muscular diseases; (ii) musculoskeletal impairments; and (iii) using drugs altering cardiorespiratory and muscular responses. Participants were informed about the study's aim and the experimental design and gave written consent to participate. The study was approved by the local ethics committee of the university (#77/20) and was conducted in accordance with the latest principles of the Declaration of Helsinki.

### 2.2 Experimental Design

Participants came to the laboratory three times, with at least 48 hours between each test. On the first day, after familiarization and anthropometric assessments, pulmonary function was evaluated. During the second session, participants underwent an incremental test to determine ${\dot{V}}_{O_{2}\;\max}$, maximal mechanical aerobic power (${\dot{W}}_{\max}$), as well as the LT on a cycle ergometer. During the third visit, the participants performed a sinusoidal protocol until exhaustion.

The experimental test took place in a climate‐controlled laboratory (constant temperature of 20 ± 1°C and relative humidity of 50 ± 5%) at a consistent time of the day to reduce potential circadian influence. On each testing day, participants were required to avoid caffeine and any other stimulant substances for at least 12 hours and to refrain from heavy exercise for at least 24 hours before the tests. SM were instructed to smoke their last cigarette 1.5 hours prior to testing, allowing for a 5%–16% reduction in blood COHb to mitigate the acute effects of cigarette smoking (McDonough and Moffatt 1999).

#### 2.2.1 Familiarization, Anthropometric and Pulmonary Function Assessment

During the first visit, participants familiarized with the equipment used for cardiopulmonary testing. Body mass and stature were measured to the nearest 0.1 kg and 1 cm, respectively, utilizing a mechanical scale with a stadiometer (Asimed, Samadell; Barcellona). On the same session, by a portable spirometer (Pony Fx, Cosmed, Rome, Italy) pulmonary function was assessed according to the criteria proposed by American Thoracic Society and the European Respiratory Society (ATS/ERS) (Berresheim et al. 2023). Maximal inspiratory and expiratory pressure (MIP and MEP, respectively) were assessed at the mouth using a portable manometer with a mouthpiece. Predicted values were determined according to Miller et al. (2005). Participants repeated the maneuver three times, and the highest value was considered.

#### 2.2.2 Incremental Exercise Test

On the second visit, ${\dot{V}}_{O_{2}\;\max}$ and ${\dot{W}}_{\max}$ were determined by a step‐wise incremental test (25 W/2 minutes) until task failure (Adami et al. 2013). Participants were asked to maintain the pedaling rate between 60 and 70 rpm. Task failure was defined as the inability to sustain the cadence for more than 5 s within the 5 rpm of the target range (Riboli et al. 2021). [La^−^] was determined at rest, at the end of each work rate, and at minute 1, 3 and 5 of recovery to assess the [La^−^] at peak. At the same time, participants were asked to indicate their rate of perceived exertion (RPE) on general (RPE_GEN_; Borg 6–20), muscular, and respiratory (RPE_MUSC_ and RPE_RESP_, respectively; CR‐10) standpoints (Borg 1982).

#### 2.2.3 Sinusoidal Protocols

During the third visit the participants performed an exhausting sinusoidal work rate at moderate intensity. The protocols involved 3 min of baseline recordings, a warm‐up of 3 min at 50 W, and 3 min at LT, after which the sinusoidal exercise began in its downward midpoint crossing toward the nadir. The mechanical power, as a function of time (*t*), fluctuated sinusoidally according to following equation:

(1) $$f\left(t\right)=\text{midpoint}+\text{amplitude}\cdot \sin\left(\frac{2\pi }{\text{Period}}t\right)$$

where, the period of the sinusoid corresponds to the duration of one full cycle of work‐rate oscillation (in minutes), midpoint is the value across which the sinusoidal function oscillates, and amplitude is the difference between midpoint and either the nadir or the zenith of the sinusoid.

**FIGURE 1:**
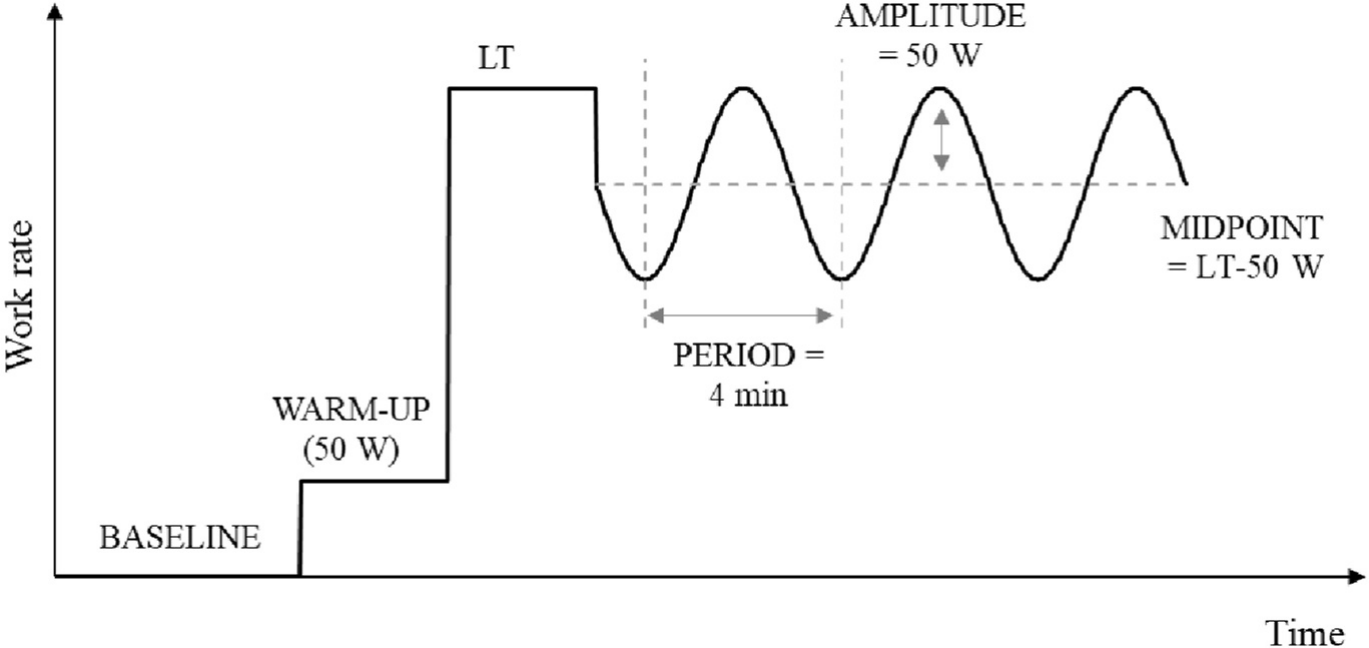
Representation of the sinusoidal protocol. LT, lactate accumulation point.

Based on preliminary test conducted in our laboratory, the sinusoidal protocol had the midpoint for power set 50 W below the LT (LT‐50_ex_), amplitude at 50 W and period at four minutes (Figure 1). We selected a period of 4 minutes to accommodate the primary component (Haouzi et al. 1993) and an amplitude of 100 W to challenge sufficiently the physiological system while remaining compatible with the selected period. Although the sinusoidal zenith approached LT, the protocol midpoint was centered 50 W below LT and thus remained predominantly within the moderate‐intensity domain (Burnley and Jones 2018) when considered in terms of overall metabolic demand.

[La^−^] and RPE values were determined at baseline, at the end of LT warm‐up, at the end of each cycle (corresponding to downward midpoint crossing after positive peak) and at minute 1, 3 and 5 of recovery.

### 2.3 Measurements

Tests were performed on an electro‐mechanically braked cycle ergometer (mod. 839E, Monark, Vansbro, Sweden). During the experiments, the work rate and cadence were continuously recorded. ${\dot{V}}_{E}$, ${\dot{V}}_{O_{2}}$, respiratory frequency ($f_{R}$), tidal volume (*V*_*T*_) and carbon dioxide production (${\dot{V}}_{{\text{CO}}_{2}}$) were measured on a breath‐by‐breath basis by a metabolic unit consisting of a turbine flowmeter, a zirconium oxygen sensor and an infrared CO_2_ meter (Quark b^2^, Cosmed, Rome, Italy). Moreover, the respiratory exchange ratio (RER) was calculated. According to manufacturer's instructions, the turbine and gas analyzers were calibrated before each test with a 3‐L syringe (mod. 5530, Hans‐Rudolph, Shawnee, KS, USA) and a certified gas mixture of known concentration (16% O_2_, 5% CO_2_, balance N_2_). HR was monitored continuously using a heart rate monitor (Polar Electro Oy, mod. S810i, Kempele, Finland). Lastly, 20 μL arterialized blood samples were collected from the ear lobe and analyzed by an enzymatic‐amperometric system (Labtrend, Bio Sensor Technology GmbH, Berlin, Germany) to determine [La^−^].

### 2.4 Data Analysis

All data were analyzed offline. The respiratory and gas exchange responses were edited of spurious breaths that resulted from swallowing, coughing, sighing or premature ending of breath, by deleting values exceeding three standard deviation (SD) from the local mean (Lamarra et al. 1987).

${\dot{V}}_{O_{2}\;\max}$ was determined as the value obtained from the plateau in the relationship between ${\dot{V}}_{O_{2}}$ and $\dot{W}$ during the incremental step‐wise test. In the event that the plateau did not occur, the highest value of ${\dot{V}}_{O_{2}}$ was considered as the maximum if: (i) HR did not increase between consecutive loads, and (ii) RER > 1.1 (Adami et al. 2013). The ${\dot{W}}_{\max}$ was defined as the mechanical work rate corresponding to the intersection between the ${\dot{V}}_{O_{2}}$ plateau and the linear relationship between ${\dot{V}}_{O_{2}}$ and $\dot{W}$.

LT was estimated by D_max_‐modified method as being correlated with maximal lactate steady‐state indicator and as considered the best approach in endurance activities (Bishop et al. 1998).

The TTE was calculated as the effective duration from the first to the last second of the sinusoidal exercise. The first 4 minutes of sinusoidal exercise were excluded from analysis to avoid any distortion in the physiological response induced by the transition of the workload intensity from LT (during the 3 min warm‐up phase) to midpoint (sinusoidal phase). The cardiorespiratory and metabolic response was fitted by the sinewave function that minimized the least squared errors through a custom‐built software (Matlab 2019b; MathWorks Inc., Natick, USA). Midpoint, amplitude, and time delays of all measured variables were calculated for each sinusoid. Time delay was determined as the time shift between the mechanical load and the physiological response. Two different time delays were determined according to the following hallmarks: (i) time delay max: maximum of the sinusoidal pattern and (ii) time delay min: minimum of the sinewave (Fukuoka et al. 1997). All these parameters were determined on a cycle‐by‐cycle basis for all the cardiorespiratory and metabolic variables to detect the presence of fatigue (Borrelli et al. 2024). Previous studies chose to overlap all the cycles performed to strengthen the analysis of the physiological response to sinusoidal exercise (Casaburi et al. 1977; Fukuoka et al. 1995, 1997; Girardi et al. 2021; Haouzi et al. 1993; Wigertz 1970). In those studies, the response to sinusoidal work rate was investigated only in protocols of relatively short duration. However, in the present protocols carried out up to exhaustion, significant differences among successive cycles were found for most of the investigated variables. This undermined the possible use of the overlapping procedure in this study. We present the cardiorespiratory and metabolic responses in relation to the percentage of total cycling duration (Supporting Information S1: Figures S1–S3).

### 2.5 Statistical Analysis

Descriptive statistics were used to define the study sample characteristics. The Shapiro–Wilk test was applied to check the normal distribution. Specifically, the differences between the two groups of cardiorespiratory and metabolic parameters during the peak of the incremental test and for the pulmonary evaluation as well as for the TTE were detected by two‐tailed unpaired Student's *t*‐test. When normality was not confirmed the Mann–Whitney *U* test was applied. The Hedge's *g* effect size with 95% confidence interval (95% CI) was also calculated and interpreted as follows: 0.00–0.19: trivial; 0.20–0.59: small; 0.60–1.19: moderate; 1.20–1.99: large; ≥ 2.00: very large (Hopkins et al. 2009). A two‐way analysis of variance for repeated measure (2‐way RM‐ANOVA) checked for differences in midpoint, amplitude, and time delays between the two groups and among sinusoidal cycles. For the statistical analysis, the minimum number of cycles completed by all subjects, minus one, and the last completed cycle were considered. For all pairwise multiple comparisons, the Bonferroni's correction was applied within each variable, adjusting for the family of comparisons performed both across cycles (first, midpoint, last) and between groups (SM vs. controls). Because the TTE was not normally distributed, the possible correlations between TTE and ${\dot{V}}_{E\;\max}$, ${\dot{V}}_{O_{2}\;\max}$, and $f_{\text{R max}}$ were assessed by Spearman's Rho coefficient (*ρ*). All statistical analyses were performed by using statistical software (IBM SPSS Statistics *v*. 29, Armonk, NY, USA). The significance level was set at *α* < 0.05. Results are presented as mean ± SD.

## 3 Results

Table 1 reported the pulmonary function outcomes. No differences in static lung volumes were identified between the two groups, but SM showed lower maximal voluntary ventilation (−8%) and higher forced expiratory time (72%).

**TABLE 1:** Respiratory function test outcomes in smokers (SM) and control group.

	Controls		SM	
	Absolute	%Predicted	Absolute	%Predicted
FVC (L)	5.8 ± 0.7	107 ± 6	5.8 ± 0.7	109 ± 9
FEV_1_ (L)	4.9 ± 0.7	109 ± 8	4.7 ± 0.6	104 ± 12
FEV_6_ (L)	5.7 ± 0.7	105 ± 8	5.7 ± 0.7	103 ± 9
FEV_1_/FVC (%)	86 ± 4	103 ± 5	82 ± 10	98 ± 11
FEF 25%–75% (L·s^−1^)	5.2 ± 0.9	100 ± 16	5.0 ± 1.4	96 ± 27
MEF75% (L·s^−1^)	8.7 ± 1.4	100 ± 11	7.9 ± 2.1	92 ± 23
MEF50% (L·s^−1^)	5.5 ± 1.2	96 ± 18	5.6 ± 1.6	99 ± 28
MEF25% (L·s^−1^)	3.0 ± 0.8	111 ± 30	2.6 ± 0.7	95 ± 27
FET 100%(s)	4.6 ± 2.3	—	7.9 ± 2.9*	—
PEF (L·s^−1^)	10.0 ± 1.7	98 ± 13	8.8 ± 1.9	86 ± 16
VRE (L)	2.0 ± 0.6	119 ± 30	2.0 ± 0.4	117 ± 20
IRV (L)	2.6 ± 0.4	—	2.4 ± 0.5	—
MVV (L·min^−1^)	184 ± 22	119 ± 7	168 ± 16*	108 ± 9*
MIP (cmH_2_O)	112 ± 14	101 ± 12	117 ± 24	105 ± 22
MEP (cmH_2_O)	120 ± 24	82 ± 17	127 ± 16	86 ± 12

*Note:* Predicted values were determined according to Miller et al. 2005 (22). Data are shown as mean ± standard deviation (SD).

Abbreviations: FEF 25%–75%, forced expiratory flow at 25% and 75% of the pulmonary volume; FET 100%, forced expiratory time; FEV_1_, forced expiratory volume during the 1st s of the test; FEV_6_, forced expiratory volume during the 6th s of the test; FVC, forced vital capacity; IRV, inspiratory reserve volume; MEF25%, instantaneous expiratory flow when 75% of FVC has to be expired; MEF50%, instantaneous expiratory flow when 50% of FVC has to be expired; MEF75%, instantaneous expiratory flow when 25% of FVC has to be expired; MEP, maximal expiratory mouth pressure; MIP, maximal inspiratory mouth pressure; MVV, maximal voluntary ventilation; PEF, peak expiratory flow; VRE, expiratory reserve volume.

^*^ *p* < 0.05 versus controls.

The principal outcomes obtained during the step‐wise incremental exercise are shown in Table 2. Despite similar HR max and ${\dot{V}}_{O_{2}\;\max}$, SM had lower ${\dot{W}}_{\max}$ (−12%; *p* = 0.029; *g* = −1.15, moderate; 95% CI = −2.20 to −0.09), ${\dot{V}}_{E\;\max}$ (−12%; *p* = 0.034; *g* = −1.13, moderate; 95% CI = −2.17 to −0.06) and $f_{R\;\max}$ (−18%; *p* = 0.024; *g* = −1.20, large; 95% CI = −2.26 to −0.13) compared to controls. Notably, no significant difference between SM and controls either in the work rate corresponding to LT (199 ± 26 vs. 219 ± 28 W for SM and controls, respectively; *p* = 0.17; *g* = −0.69, moderate; 95% CI = −1.70–0.32) or in the [La^−^] (3.68 ± 1.13 vs. 3.36 ± 1.12 mmol·L^−1^ for SM and controls, respectively; *p* = 0.39; *g* = −0.42, small; 95% CI = −1.41–0.57) or in the ${\dot{V}}_{O_{2}}$ (2844 ± 428 vs. 2937 ± 339 mL·min^−1^ for SM and controls, respectively; *p* = 0.64; *g* = −0.23, small; 95% CI = −1.21–0.76) were found.

**TABLE 2:** Main outcomes of step‐wise incremental test at peak of exercise.

	Controls	SM
$\dot{W}$ (W)	278 ± 29	246 ± 24*
${\dot{V}}_{O_{2}}$ (mL·min^−1^)	3623 ± 302	3385 ± 341
${\dot{V}}_{CO_{2}}$ (mL·min^−1^)	3870 ± 334	3815 ± 396
RER	1.07 ± 0.02	1.13 ± 0.05*
HR (beats·min^−1^)	184 ± 10	185 ± 10
${\dot{V}}_{E}$ (L·min^−1^)	142 ± 16	125 ± 12*
$f_{R}$ (breaths·min^−1^)	58 ± 10	47 ± 7*
$V_{T}$ (L)	2.51 ± 0.52	2.67 ± 0.40
[La^−^] (mM)	9.7 ± 2.2	9.3 ± 1.2
RPE GENERAL (a.u.)	19 ± 1	19 ± 1
RPE MUSCULAR (a.u.)	10 ± 1	10 ± 1
RPE RESPIRATORY (a.u.)	9 ± 1	10 ± 1

*Note:* Maximal mechanical aerobic power ($\dot{W}$), pulmonary oxygen uptake (${\dot{V}}_{O_{2}}$), carbon dioxide production (${\dot{V}}_{{\text{CO}}_{2}}$), respiratory exchange ratio (RER), heart rate (HR), pulmonary ventilation (${\dot{V}}_{E}$), respiratory rate ($f_{R}$), tidal volume ($V_{T}$), blood lactate concentration ([La^−^]), rates of perceived exertion on a general, respiratory and muscular standpoints. Data are shown as mean ± standard deviation (SD).

^*^ *p* < 0.05 versus controls.

### 3.1 Sinusoidal Protocol

**FIGURE 2:**
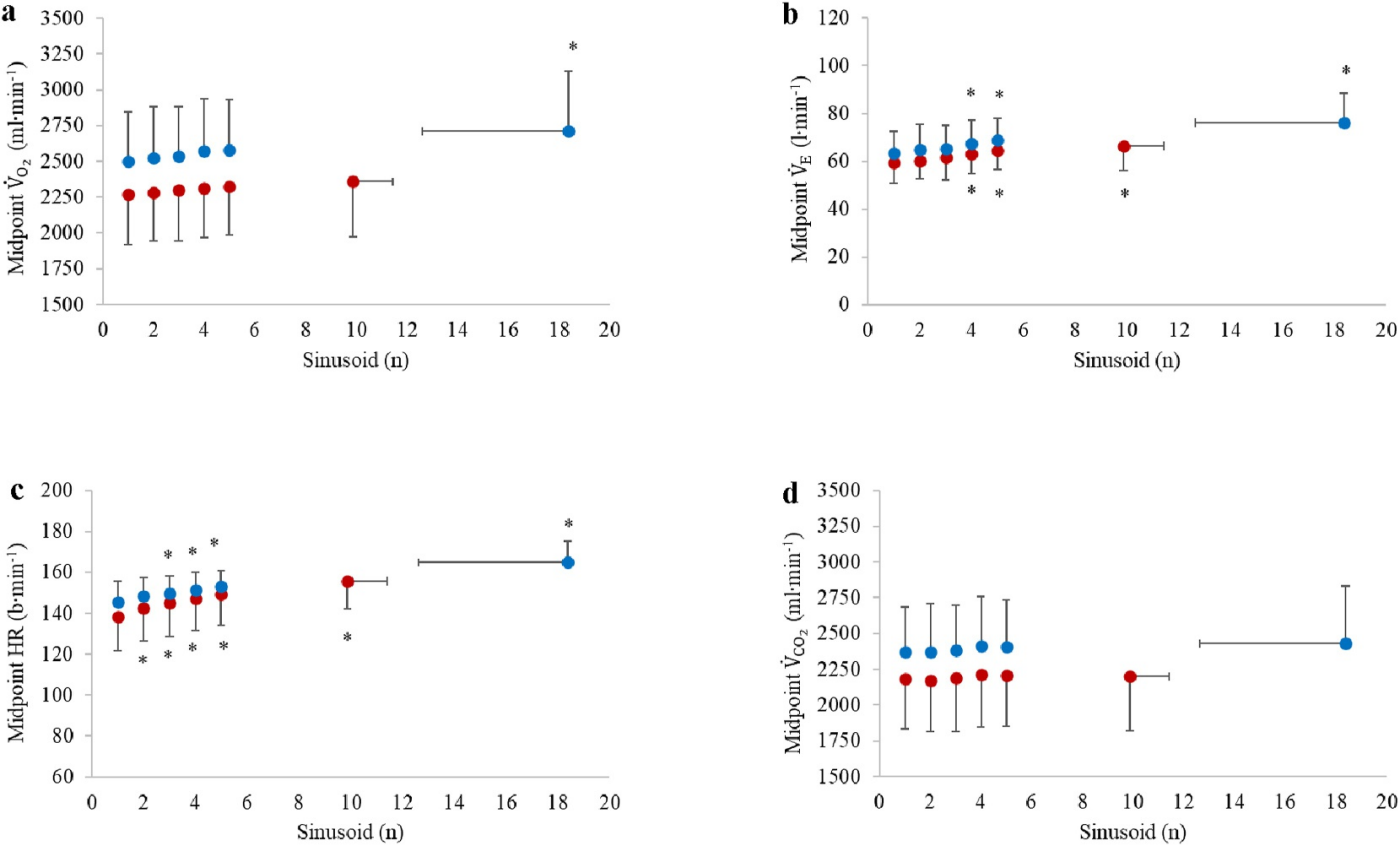
Midpoint response of pulmonary oxygen uptake (${\dot{V}}_{O_{2}}$, panel a), ventilation (${\dot{V}}_{E}$, panel b), heart rate (HR, panel c), carbon dioxide production (${\dot{V}}_{{CO}_{2}}$, panel d) to each sinusoid in controls (blue circles) and smokers (red circles). *p < 0.05 versus sinusoid 1; ^#^ *p* < 0.05 versus controls. Data are shown as mean ± standard deviation (SD).

**FIGURE 3:**
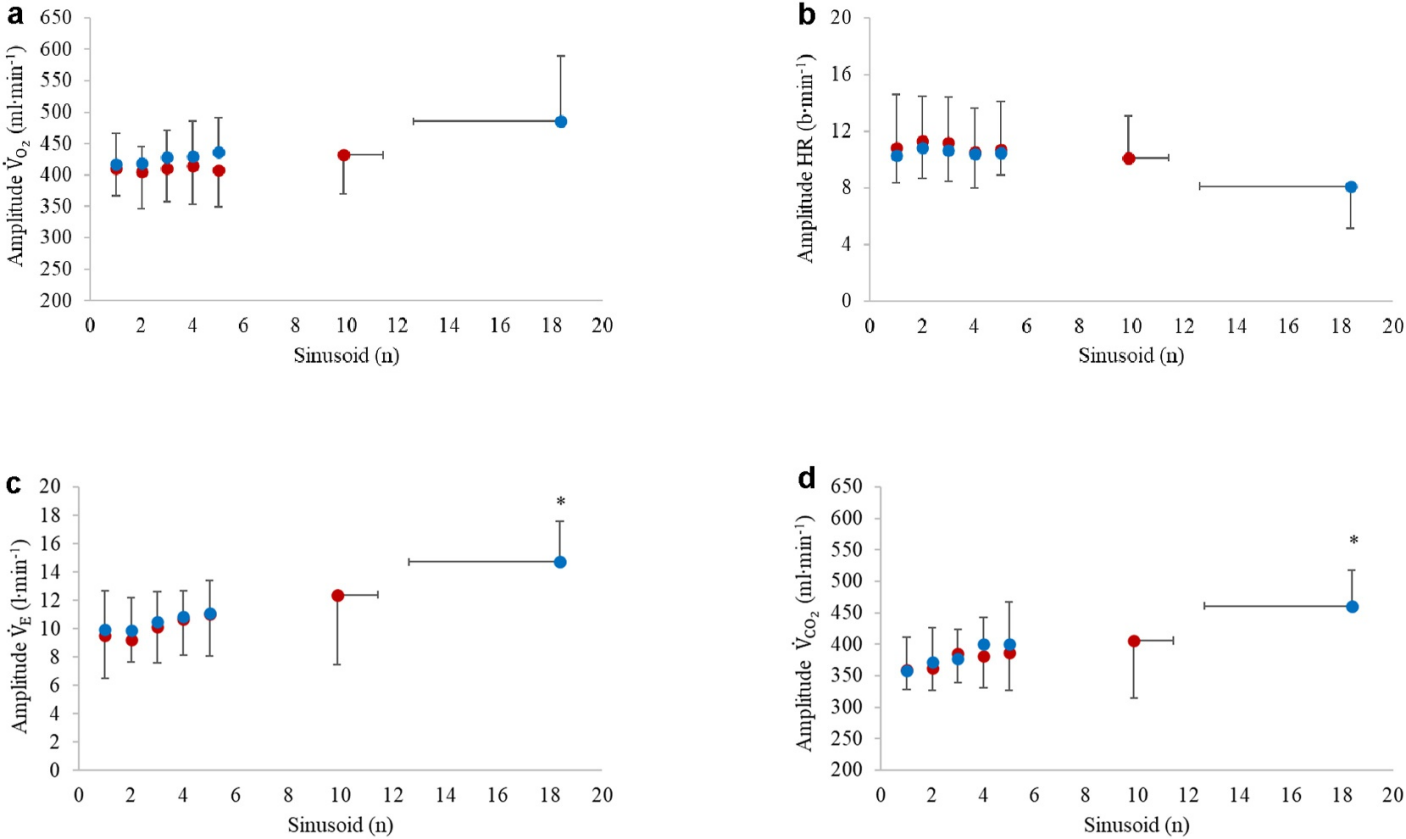
Amplitude response between midpoint and zenith of pulmonary oxygen uptake (${\dot{V}}_{O_{2}}$, panel a), ventilation (${\dot{V}}_{E}$, panel b), heart rate (HR, panel c), carbon dioxide production (${\dot{V}}_{{CO}_{2}}$, panel d) to each sinusoid in controls (blue circles) and smokers (red circles). *p < 0.05 versus sinusoid 1; ^#^ *p* < 0.05 versus controls. Data are shown as mean ± standard deviation (SD).

**FIGURE 4:**
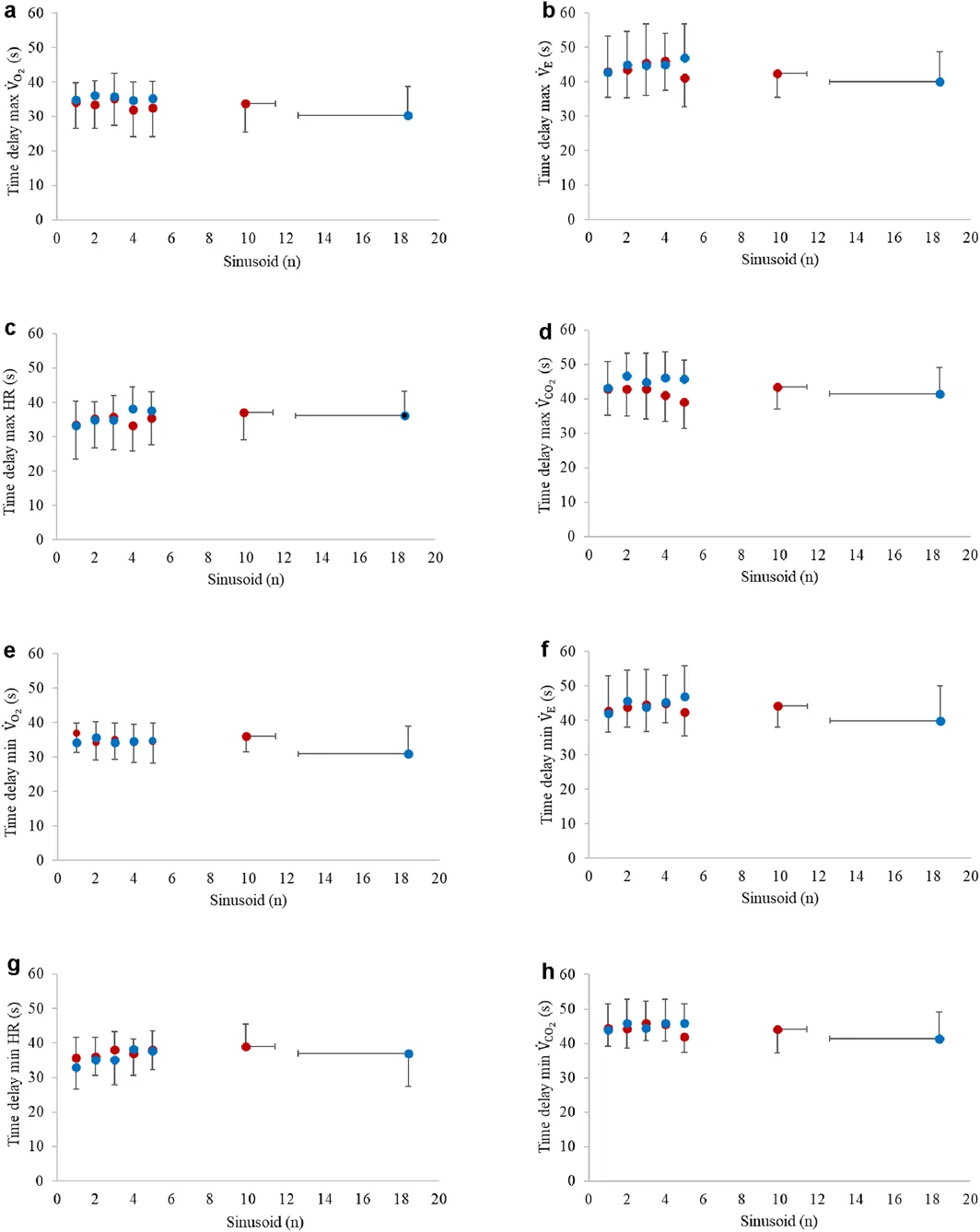
Time delay at the maximum (max) and the minimum of the sinewave (min) of pulmonary oxygen uptake (${\dot{V}}_{O_{2}}$, panel a and e for t_D max_ and t_D min_, respectively), ventilation (${\dot{V}}_{E}$, panel b and f for t_D max_ and t_D min_, respectively), heart rate (HR, panel c and g for t_D max_ and t_D min_, respectively), carbon dioxide production (${\dot{V}}_{{CO}_{2}}$, panel d and h for t_D max_ and t_D min_, respectively) to each sinusoid in controls (blue circles) and smokers (red circles). *p < 0.05 versus sinusoid 1; ^#^ *p* < 0.05 versus controls. Data are shown as mean ± standard deviation (SD).

For TTE normality was not confirmed, requiring the Mann–Whitney *U* test. TTE of SM was lower than controls (2461 ± 386 vs. 4512 ± 1359 s for SM and controls, respectively; *p* = 0.001; *g* = −1.96, large, CI_95%_: −3.13 to −0.75). The main results of the sinusoidal variables are reported in Figures 2, 3, 4.

### 3.2 Midpoint

No differences in midpoint of all the cardiorespiratory and metabolic parameters were reported between SM and controls. Regarding the within‐group analysis, in controls, the midpoint of the last cycle of ${\dot{V}}_{O_{2}}$ was higher compared to the first one (*p* = 0.001), whereas in SM it remained constant. In both SM and controls, HR midpoint showed an increase starting from the second cycle (*p* < 0.01), whereas in ${\dot{V}}_{E}$ midpoint exhibited a rise from the fourth cycle onward (*p* < 0.05). However, in both groups, ${\dot{V}}_{{\text{CO}}_{2}}$ midpoint remained constant. Statistical details are shown in Supporting Information S2: Tables S1 and S3.

### 3.3 Amplitude

No differences in amplitude of the cardiorespiratory and metabolic parameters were observed between SM and controls. In both groups, the amplitude ${\dot{V}}_{O_{2}}$ and HR remained stable throughout the entire duration of the protocol. Conversely, only in controls, the amplitude of ${\dot{V}}_{E}$ and ${\dot{V}}_{CO_{2}}$ was higher in the last cycle compared to the first one (*p* = 0.003, *p* = 0.048 for ${\dot{V}}_{E}$ and ${\dot{V}}_{CO_{2}}$, respectively). Statistical details are shown in Supporting Information S2: Tables S1 and S3.

### 3.4 Time Delays

No differences in cardiorespiratory and metabolic time delays were observed either between the two groups nor between cycles.

### 3.5 Blood Lactate Concentration [La^−^] and Rate of Perceived Exertion (RPE)

**FIGURE 5:**
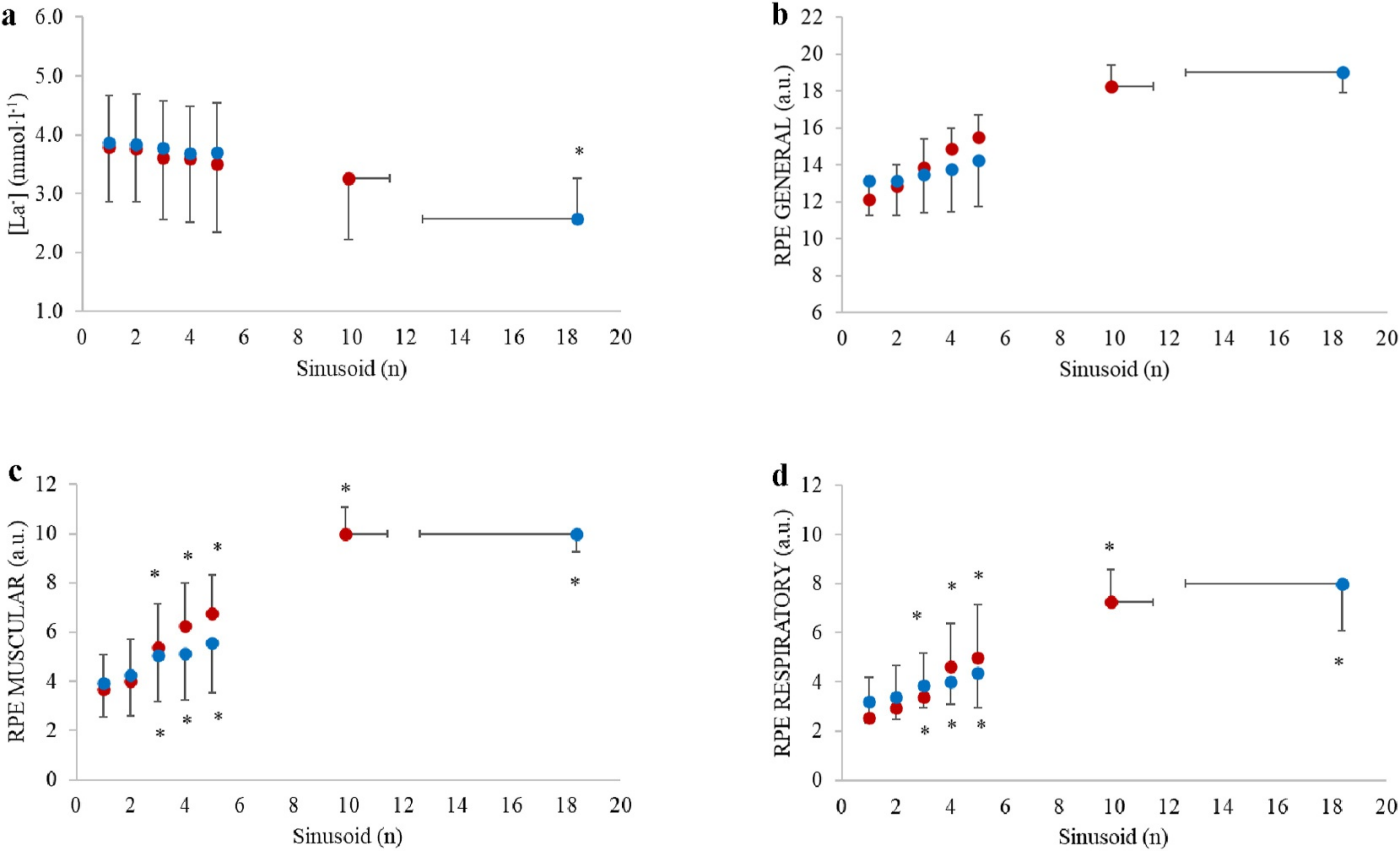
Blood lactate concentration ([La^−^], panel a), rate of perceived exertion on general (RPE GENERAL, panel b), muscular (RPE MUSCULAR, panel c) and respiratory (RPE RESPIRATORY, panel d) standpoint to each sinusoid in controls (blue circles) and smokers (red circles). *p < 0.05 versus sinusoid 1; ^#^ *p* < 0.05 versus controls. Data are shown as mean ± standard deviation (SD).

As shown in Figure 5, [La^−^] values were similar between SM and controls. In SM, [La^−^] remained stable throughout the protocol, whilst in controls, [La^−^] in the last cycle was lower than in the first one (*p* < 0.001). No differences in RPE values were observed between SM and controls. In both groups, RPE GENERAL, MUSCULAR and RESPIRATORY were greater in the last cycle compared to the first one (*p* < 0.001). Statistical details are shown in Supporting Information S2: Tables S2 and S4.

### 3.6 Correlations

**FIGURE 6:**
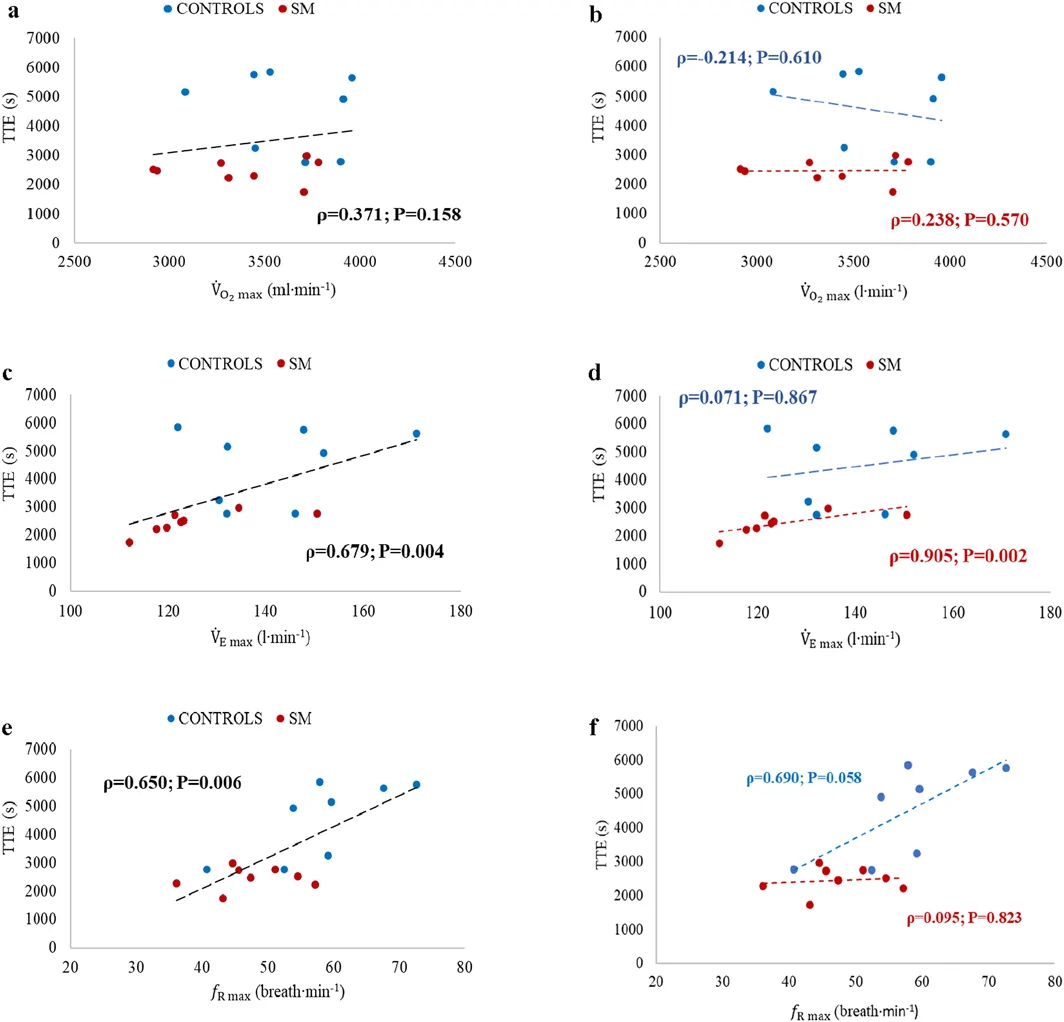
Correlations between time to exhaustion during sinusoidal protocol (TTE) and maximum pulmonary oxygen uptake (${\dot{V}}_{O_{2}\;\max}$) shown as pooled correlations (panel a) and divided by group (panel b). Correlations between TTE and ventilation (${\dot{V}}_{E\;\max}$) are shown as pooled (panel c) and group‐specific (panel d) relationships. Correlations between TTE and respiratory frequency ($f_{R\;\max}$), presented as pooled (panel e) and divided by group (panel f).

Correlations between TTE and ${\dot{V}}_{O_{2}\;\max}$, ${\dot{V}}_{E\;\max}$ and $f_{R\;\max}$ were shown in Figure 6. Moreover, the correlation between smoking exposure (pack‐years) and time to exhaustion was not significant (*ρ* = −0.419; *p* = 0.301).

## 4 Discussion

As hypothesized, SM exhibited a shorter TTE, indicating that cigarette smoking affects negatively exercise tolerance even in young individuals with a relatively short smoking history and no diagnosed lung or cardiovascular disease. Surprisingly, this difference was not accompanied by differences in midpoint, amplitude, and time delays of any cardiorespiratory, metabolic, or perceptual parameters. However, SM did not exhibit an increase in ${\dot{V}}_{O_{2}}$ and ${\dot{V}}_{E}$ midpoint as in controls throughout the exhaustion exercise.

### 4.1 Preliminary Considerations

In line with previous studies on young SM (Borrelli et al. 2025; Lorensia et al. 2021), no differences in static lung volumes, in forced vital capacity (FVC), in forced expiratory volume during the 1st s (FEV_1_), and the FEV_1_/FVC ratio were observed between groups. This was attributed to their short smoking history and to their relatively small number of cigarettes smoked per day. Indeed, the deterioration of lung function in young SM has been directly related in previous studies to daily cigarette consumption (Elbehairy et al. 2016; Melliti et al. 2021). Furthermore, SM confirmed their lower maximal voluntary ventilation and forced expiratory time during dynamic pulmonary function tests, indicating a reduction in respiratory endurance (Graham et al. 2019). The absence of a correlation between smoking exposure (measured in pack‐years) and time to exhaustion suggests that a dose–response relationship cannot be hypothesized.

### 4.2 Effect of Cigarette Smoking on Sinusoidal Protocol Response

As hypothesized, the sinusoidal protocol duration was shorter in SM compared to controls. On this topic, available data are limited to Bruce protocol tests conducted in young individuals (Papathanasiou et al. 2007; Sidney et al. 1993) and middle‐aged SM (Blair et al. 1985), which are not comparable to the type of exercise protocol adopted in the present study. The reduction in exercise duration was attributed to acute and chronic effects of cigarette smoking, including COHb formation and increased airway resistance as well as long‐term cardiovascular and pulmonary system damage (de Tarso Muller et al. 2019; McDonough and Moffatt 1999). Moreover, the previously reported lower physical activity level in SM (Blair et al. 1985) cannot explain our results, as SM and controls were matched for physical activity level. This result is particularly surprising given the absence of between‐group differences in cardiorespiratory and metabolic sinusoidal parameters (i.e., midpoint, amplitude, time delay), which is in agreement only in part with our hypothesis.

The lack of difference in midpoint for all cardiorespiratory and metabolic parameters between groups could be ascribed to the fact that the sinusoidal work rate midpoint was individualized at the relative LT work rate. This finding aligns with previous studies reporting no differences in cardiorespiratory and metabolic responses to submaximal exercise (Borrelli et al. 2025; Hirsch et al. 1985). Despite no differences emerging between groups at any time point, the lack of an increase in ${\dot{V}}_{O_{2}}$ and ${\dot{V}}_{E}$ midpoint observed in SM over the course of the protocol is noteworthy. In parallel with a progressive rise in ${\dot{V}}_{E}$, the ${\dot{V}}_{O_{2}}$ increase observed in controls, although modest in magnitude (∼250–300 mL·min^−1^), exceeded the minimal drift typically reported during prolonged moderate‐intensity exercise (Jones et al. 2011), indicating a growing global physiological cost over time. Nevertheless, it did not correspond to a classical ${\dot{V}}_{O_{2}}$ slow component, as described during heavy or severe constant‐load exercise, which is larger in magnitude (Jones et al. 2011). Therefore, it remains plausible that exercise in SM ended before these time‐dependent compensatory responses could fully develop. The absence of a progressive rise in ${\dot{V}}_{O_{2}}$ midpoint in SM likely reflects a reduced tolerance to the progressive metabolic and ventilatory stress associated with prolonged exhausting exercise, leading to earlier task failure and constraining the development of time‐dependent physiological adjustments. This interpretation is further supported by the correlation analysis, which revealed no association between ${\dot{V}}_{O_{2}\;\max}$ and TTE but significant positive relationships between TTE and ${\dot{V}}_{E\;\max}$ only in SM. These findings suggest that ventilatory capacity, and possibly respiratory muscle work, may represent a limiting factor for exercise tolerance in SM (Baty et al. 2016). Respiratory and metabolic impairments have already been demonstrated in our previous papers (Borrelli et al. 2025, 2026), in which SM exhibited slower cardiorespiratory and metabolic kinetics during moderate exercise and lower ${\dot{V}}_{O_{2}\;\max}$, ${\dot{W}}_{\max}$, and ${\dot{V}}_{E\;\max}$ at the peak of the incremental test. Notably, these alterations were again observed in young SM without any clinically evident cardiorespiratory disease or symptoms.

The similar progression of perceived exertion between groups indicates that premature termination in SM was not related to altered perceptual responses, but rather to physiological limitations emerging below conscious perception.

The lack of difference between groups for time delays in all the investigated variables of the sinusoidal exercise may be explained by the duration of the period. Indeed, Davies et al. (2017) demonstrated that shorter work: recovery cycle duration ratios were associated with a greater contribution of substrate‐level phosphorylation for muscular ATP synthesis, specifically through increased PCr breakdown. This could represent a limitation for SM. However, the use of a 4‐min period in the present study appears to have mitigated this potential limitation. The absence of differences in time delays between SM and controls may also explain the lack of the difference in amplitude of all cardiorespiratory and metabolic parameters. Indeed, in case of a longer time delays in SM, blunted amplitude values would have been observed due to the inability of the systems to promptly adapt to the control level. The absence of studies investigating the impact of cardiorespiratory limitations on response to sinusoidal work rate makes direct comparisons difficult. Further research using different periods in clinical populations is needed.

Although our findings suggest an impaired ventilatory and metabolic response to exhaustive exercise in SM, the potential contribution of peripheral mechanisms should also be considered. In fact, cigarette smoking‐induced alterations in skeletal muscle oxygenation, characterized by reduced capillary density and fiber cross‐sectional area (de Tarso Muller et al. 2019; Degens et al. 2015), may contribute to the reduced TTE. However, as peripheral muscle function and tissue oxygenation were not directly assessed, the present study cannot determine the extent to which smoking‐related skeletal muscle alterations induced exercise intolerance.

### 4.3 Study Limitations

This study has some known limitations. Firstly, it does not include measurements of plethysmographic lung volumes, lung diffusion capacity, or muscle‐level O_2_ extraction, which would provide a more comprehensive understanding of the heart‐lung‐muscle interaction. Secondly, the sample consists exclusively of male participants, limiting generalizability. In fact, given the differences in cardiorespiratory and metabolic response to exercise and respiratory function between females and males (Diaz‐Canestro et al. 2022; Harms and Rosenkranz 2008; Santisteban et al. 2022) and the effect of menstrual cycle phase on cardiorespiratory and metabolic response (Barba‐Moreno et al. 2022; Janse de Jonge 2003), we chose to minimize the variance between the two groups by focusing exclusively on males, in order to better highlight the alterations induced by cigarette smoking. The variability in cigarette consumption and the relatively small sample size may limit the ability to definitively exclude a potential association between smoking exposure and TTE. Lastly, although a 1.5‐h abstinence period prior to testing was requested to reduce acute smoking effects, the absence of direct COHb measurements does not allow verification of its effectiveness.

## 5 Conclusion

Cigarette smoking reduced exercise tolerance in young physically active SM with no known lung or cardiovascular disease. These findings suggest a potential contribution of ventilatory and metabolic limitation. Overall, our results indicate that exercise intolerance in SM may emerge even in the absence of detectable alterations in central cardiorespiratory dynamics during sinusoidal exercise. Further research is warranted to better elucidate the relative contribution of peripheral mechanisms. This apparent dissociation between preserved dynamic responses and reduced exercise tolerance, despite comparable respiratory and metabolic responses at exhaustion between groups, represents a pivotal finding of the present study, offering important insight into the mechanisms underlying early exercise intolerance in SM.

### 5.1 Future Perspectives and Clinical Implications

The findings of this study have important implications in public health by clarifying the detrimental effects of cigarette smoking on cardiorespiratory and metabolic responses during exercise also in young SM. These alterations may act as an early warning signal, indicating that even asymptomatic, physically active, young SM with a short cigarette smoking history already exhibits impairments in exercise capacity. Highlighting such early dysfunction could strengthen preventive strategies and motivate current SM to quit or discourage potential SM from initiating this habit, thereby improving individual physical, mental, and social well‐being and reducing the societal burden of cigarette smoking‐related diseases.

## Author Contributions

M.B. and F.E. conceived and designed research, M.B., A.M., N.T., E.L. and S.R. performed experiments, M.B., A.M. and S.R. analyzed data, M.B., S.R., S.L, G.C. and E.C. interpreted results of experiments, M.B. prepared figures, M.B. and F.E. drafted manuscript, M.B, E.C., S.R., and F.E. edited and revised manuscript. All authors approved final version of the manuscript.

## Funding

This research project was supported by institutional grant provided by the Department of Biomedical Sciences for Health, Università degli Studi di Milano.

## Consent

The study received approval from the ethics committee of the Università degli Studi di Milano (#77/20).

## Conflicts of Interest

The authors declare no conflicts of interest.

## Supporting information


Supporting Information S1

Supporting Information S2

## Data Availability

The data that support the findings of this study are available on request from the corresponding author. The data are not publicly available due to privacy or ethical restrictions.
