# Supplementary material for: Exercise Intolerance in Young Physically Active Smokers Compared to Non‐Smokers During Sinusoidal Cycling
Source: Eur J Sport Sci. 2026 Aug 24;26(9):e70238. doi: 10.1002/ejsc.70238 (PMC13501398; doi:10.1002/ejsc.70238)
Supplement: Supplementary file 1 [file EJSC-26-e70238-s001.docx]

**Exercise intolerance in young physically active smokers compared to non-smokers during sinusoidal cycling**

**Running title: Exercise intolerance in young smokers**

**Figure S1:** Midpoint response of pulmonary oxygen uptake ($\dot{V}_{O_{2}}$, panel a), ventilation ($\dot{V}_{E}$, panel b), heart rate (HR, panel c), carbon dioxide production ($\dot{V}_{{CO}_{2}}$, panel d) to each sinusoid in controls (blue circles) and smokers (red circles). *p<0.05 vs sinusoid 1; ^#^p<0.05 vs controls. Data are shown as mean ± standard deviation (SD)

**a**

**b**

**d**

**c**

**Figure S2:** Amplitude response between midpoint and zenith of pulmonary oxygen uptake ($\dot{V}_{O_{2}}$, panel a), ventilation ($\dot{V}_{E}$, panel b), heart rate (HR, panel c), carbon dioxide production ($\dot{V}_{{CO}_{2}}$, panel d) to each sinusoid in controls (blue circles) and smokers (red circles). *p<0.05 vs sinusoid 1; ^#^p<0.05 vs controls. Data are shown as mean ± standard deviation (SD)

**a**

**b**

**c**

**d**

**Fig. S3** Time delay at the maximum (max) and the minimum of the sinewave (min) of pulmonary oxygen uptake ($\dot{V}_{O_{2}}$, panel a and e for t_D max_ and t_D min_, respectively), ventilation ($\dot{V}_{E}$, panel b and f for t_D max_ and t_D min_, respectively), heart rate (HR, panel c and g for t_D max_ and t_D min_, respectively), carbon dioxide production ($\dot{V}_{{CO}_{2}}$, panel d and h for t_D max_ and t_D min_, respectively) to each sinusoid in controls (blue circles) and smokers (red circles). *p<0.05 vs sinusoid 1; ^#^p<0.05 vs controls. Data are shown as mean ± standard deviation (SD)

**g**

**h**

**f**

**e**

**d**

**c**

**a**

**b**
