# Supplementary material for: Exercise Intolerance in Young Physically Active Smokers Compared to Non‐Smokers During Sinusoidal Cycling
Source: Eur J Sport Sci. 2026 Aug 24;26(9):e70238. doi: 10.1002/ejsc.70238 (PMC13501398; doi:10.1002/ejsc.70238)
Supplement: Supplementary file 2 [file EJSC-26-e70238-s002.docx]

**Exercise intolerance in young physically active smokers compared to non-smokers during sinusoidal cycling**

**Running title: Exercise intolerance in young smokers**

**Table S1:** Between groups effect in midpoint, amplitude, time delay at maximum and at minimum of the sinusoidal pattern response of pulmonary oxygen uptake ($\dot{V}_{O_{2}}$), ventilation ($\dot{V}_{E}$), carbon dioxide production ($\dot{V}_{{CO}_{2}}$) and heart rate (HR) to each sinewave in controls and smokers. Data are shown as mean ± standard deviation (SD). *p<0.05 vs sinusoid 1.

| **Parameters** | **CONTROLS** | | | | | |  | **SMOKERS** | | | | | |
| --- | --- | --- | --- | --- | --- | --- | --- | --- | --- | --- | --- | --- | --- |
|  | **Sinusoid 1** | **Sinusoid 2** | **Sinusoid 3** | **Sinusoid 4** | **Sinusoid 5** | **Sinusoid Last** |  | **Sinusoid 1** | **Sinusoid 2** | **Sinusoid 3** | **Sinusoid 4** | **Sinusoid 5** | **Sinusoid Last** |
|  |  |  |  |  |  |  |  |  |  |  |  |  |  |
| **Midpoint** ${\dot{\mathbf{V}}}_{\mathbf{O}_{\mathbf{2}}}$ **(ml∙min^-1^)** | 2497 ± 347 | 2523 ± 357 | 2536 ± 343 | 2573 ± 362 | 2578 ± 355 | 2710 ± 420 |  | 2271 ± 354  P=0.218  g=0.61  CI_95%_=-0.39-1.61 | 2281 ± 341  P=0.187  g=0.66  CI_95%_=-0.35-1.66 | 2303 ± 363  P=0.209  g=0.62  CI_95%_=-0.38-1.63 | 2315 ± 351  P=0.169  g=0.68  CI_95%_=-0.32-1.69 | 2324± 342  P=0.168  g=0.69  CI_95%_=-0.32-1.70 | 2358 ± 388  P=0.103  g=0.82  CI_95%_=-0.20-1.84 |
| **Midpoint** ${\dot{\mathbf{V}}}_{\mathbf{E}}$ **(l∙min^-1^)** | 63 ± 9 | 65 ± 10 | 65 ± 10 | 67 ± 10 | 69 ± 9 | 76 ± 12 |  | 60 ± 9  P=0.418  g=0.40  CI_95%_=-0.59-1.39 | 60 ± 8  P=0.322  g=0.48  CI_95%_=-0.51-1.48 | 62 ± 9  P=0.454  g=0.37  CI_95%_=-0.62-1.35 | 63 ± 8  P=0.348  g=0.46  CI_95%_=-0.53-1.45 | 64 ± 8  P=0.323  g=0.49  CI_95%_=-0.51-1.48 | 66 ± 10  P=0.107  g=0.82  CI_95%_=-0.20-1.84 |
| **Midpoint** ${\dot{\mathbf{V}}}_{\mathbf{C}\mathbf{O}_{\mathbf{2}}}$ **(ml∙min^-1^)** | 2374 ± 310 | 2370 ± 338 | 2382 ± 318 | 2411 ± 350 | 2410 ± 327 | 2431 ± 400 |  | 2184 ± 350  P=0.270  g=0.54  CI_95%_=-0.46-1.54 | 2170 ± 353  P=0.267  g=0.55  CI_95%_=-0.46-1.54 | 2189 ± 372  P=0.283  g=0.53  CI_95%_=-0.47-1.52 | 2214 ± 370  P=0.291  g=0.52  CI_95%_=--0.48-1.51 | 2208 ± 355  P=0.256  g=0.56  CI_95%_=-0.44-1.56 | 2200 ± 380  P=0.255  g=0.56  CI_95%_=-0.44-1.56 |
| **Midpoint HR (b∙min^-1^)** | 145 ± 10 | 148 ± 9 | 150 ± 9 | 152 ± 8 | 153 ± 8 | 165 ± 10 |  | 138 ± 16  P=0.320  g=0.49  CI_95%_=-0.50-1.49 | 142 ± 16  P=0.374  g=0.43  CI_95%_=-0.56-1.42 | 145 ± 16  P=0.483  g=0.35  CI_95%_=-0.64-1.33 | 147 ± 16  P=0.511  g=0.34  CI_95%_=-0.65-1.33 | 149 ± 15  P=0.513  g=0.31  CI_95%_=-0.67-1.30 | 156 ± 14  P=0.152  g=0.72  CI_95%_=-0.29-1.73 |
|  |  |  |  |  |  |  |  |  |  |  |  |  |  |
| **Amplitude** ${\dot{\mathbf{V}}}_{\mathbf{O}_{\mathbf{2}}}$ **(ml∙min^-1^)** | 418 ± 44 | 418 ± 59 | 428 ± 53 | 430 ± 62 | 436 ± 59 | 485 ± 62 |  | 411 ± 48  P=0.753  g=0.15  CI_95%_=-0.83-1.13 | 405 ± 26  P=0.580  g=0.27  CI_95%_=-0.72-1.25 | 411 ± 42  P=0.483  g=0.34  CI_95%_=-0.65-1.33 | 415 ± 56  P=0.609  g=0.25  CI_95%_=-0.74-1.23 | 408 ± 56  P=0.344  g=0.46  CI_95%_=-0.53-1.46 | 432 ±104  P=0.231  g=0.59  CI_95%_=-0.41-1.59 |
| **Amplitude** ${\dot{\mathbf{V}}}_{\mathbf{E}}$ **(l∙min^-1^)** | 10 ± 3 | 10 ± 2 | 10 ± 2 | 11 ± 2 | 11 ± 2 | 15 ± 3 |  | 10 ± 3  P=0.766  g=0.14  CI_95%_=-0.84-1.13 | 9 ± 2  P=0.514  g=0.32  CI_95%_=-0.67-1.30 | 10 ± 3  P=0.773  g=0.14  CI_95%_=-0.84-1.12 | 11 ± 3  P=0.859  g=0.09  CI_95%_=-0.89-1.07 | 11 ± 3  P=0.967  g=0.02  CI_95%_=-0.96-1.00 | 12 ± 5  P=0.264  g=0.55  CI_95%_=-0.45-1.55 |
| **Amplitude** ${\dot{\mathbf{V}}}_{\mathbf{C}\mathbf{O}_{\mathbf{2}}}$ **(ml∙min^-1^)** | 358 ± 53 | 372 ± 53 | 377 ± 47 | 400 ± 42 | 401 ± 67 | 461 ± 56 |  | 359 ± 31  P=0.982  g=-0.01  CI_95%_=-0.99-0.97 | 363 ± 36  P=0.679  g=0.20  CI_95%_=-0.78-1.18 | 386 ± 47  P=0.712  g=-0.18  CI_95%_=-1.16-0.80 | 381 ± 50  P=0.428  g=0.39  CI_95%_=--0.60-1.38 | 386 ± 60  P=0.663  g=0.21  CI_95%_=-0.77-1.19 | 406 ± 91  P=0.169  g=0.69  CI_95%_=-0.32-1.69 |
| **Amplitude HR (b∙min^-1^)** | 10 ± 2 | 11 ± 2 | 11 ± 2 | 10 ± 2 | 10 ± 2 | 8 ± 3 |  | 11 ± 4  P=0.713  g=-0.18  CI_95%_=-1.16-0.80 | 11 ± 3  P=0.718  g=-0.17  CI_95%_=-1.16-0.81 | 11 ± 3  P=0.709  g=-0.18  CI_95%_=-1.16-0.80 | 11 ± 3  P=0.945  g=-0.03  CI_95%_=-1.01-0.95 | 11 ± 3  P=0.850  g=-0.09  CI_95%_=-1.07-0.89 | 10 ± 3  P=0.194  g=-0.64  CI_95%_=-1.65-0.36 |
|  |  |  |  |  |  |  |  |  |  |  |  |  |  |
| **Time delay max** ${\dot{\mathbf{V}}}_{\mathbf{O}_{\mathbf{2}}}$ **(s)** | 35 ± 5 | 36 ± 4 | 36 ± 7 | 35 ± 5 | 35 ± 5 | 30 ± 8 |  | 34 ± 8  P=0.849  g=0.09  CI_95%_=-0.89-1.07 | 33 ± 7  P=0.346  g=0.46  CI_95%_=-0.53-1.45 | 35 ± 8  P=0.865  g=0.08  CI_95%_=-0.90-1.06 | 32 ± 8  P=0.423  g=0.39  CI_95%_=-0.60-1.38 | 33 ± 8  P=0.463  g=0.36  CI_95%_=-0.63-1.34 | 34 ± 8  P=0.430  g=-0.38  CI_95%_=-1.37-0.60 |
| **Time delay max** ${\dot{\mathbf{V}}}_{\mathbf{E}}$ **(s)** | 43 ± 10 | 45 ± 10 | 45 ± 12 | 45 ± 9 | 47 ± 10 | 40 ± 9 |  | 43 ± 7  P=0.978  g=-0.01  CI_95%_=-0.99-0.97 | 44 ± 8  P=0.743  g=0.16  CI_95%_=-0.82-1.14 | 46 ± 10  P=0.909  g=-0.06  CI_95%_=-1.04-0.93 | 46 ± 8  P=0.823  g=-0.11  CI_95%_=-1.09-0.87 | 41 ± 8  P=0.217  g=0.61  CI_95%_=-0.39-1.61 | 42 ± 7  P=0.556  g=-0.29  CI_95%_=-1.27-0.70 |
| **Time delay max** ${\dot{\mathbf{V}}}_{\mathbf{C}\mathbf{O}_{\mathbf{2}}}$ **(s)** | 43 ± 8 | 47 ± 7 | 45 ± 8 | 46 ± 7 | 46 ± 5 | 42 ± 8 |  | 43 ± 8  P=0.978  g=0.05  CI_95%_=-0.93-1.03 | 43 ± 8  P=0.743  g=0.51  CI_95%_=-0.49-1.50 | 43 ± 9  P=0.909  g=0.22  CI_95%_=-0.76-1.20 | 41 ± 8  P=0.823  g=0.64  CI_95%_=-0.36-1.65 | 39 ± 8  P=0.217  g=0.96  CI_95%_=-0.08-1.99 | 44 ± 6  P=0.556  g=-0.27  CI_95%_=-1.25-0.71 |
| **Time delay max HR (s)** | 33 ± 7 | 35 ± 5 | 35 ± 7 | 38 ± 6 | 38 ± 5 | 36 ± 7 |  | 34 ± 10  P=0.955  g=-0.03  CI_95%_=-1.01-0.95 | 35 ± 9  P=0.891  g=-0.07  CI_95%_=-1.05-0.91 | 36 ± 10  P=0.838  g=-0.10  CI_95%_=-1.08-0.88 | 33 ± 7  P=0.172  g=0.68  CI_95%_=-0.33-1.69 | 36 ± 8  P=0.542  g=0.30  CI_95%_=-0.69-1.28 | 37 ± 8  P=0.796  g=-0.12  CI_95%_=-1.11-0.86 |
|  |  |  |  |  |  |  |  |  |  |  |  |  |  |
| **Time delay min** ${\dot{\mathbf{V}}}_{\mathbf{O}_{\mathbf{2}}}$ **(s)** | 34 ± 5 | 36 ± 5 | 34 ± 6 | 35 ± 5 | 35 ± 5 | 31 ± 8 |  | 37 ± 6  P=0.34  g=-0.46  CI_95%_=-1.46-0.53 | 34 ± 5  P=0.541  g=0.30  CI_95%_=-0.69-1.28 | 35 ± 6  P=0.734  g=-0.16  CI_95%_=-1.15-0.82 | 34 ± 6  P=0.852  g=0.09  CI_95%_=-0.89-1.07 | 34 ± 6  P=0.932  g=0.04  CI_95%_=-0.94-1.02 | 36 ± 5  P=0.142  g=-0.74  CI_95%_=-1.75-0.28 |
| **Time delay min** ${\dot{\mathbf{V}}}_{\mathbf{E}}$ **(s)** | 42 ± 11 | 46 ± 9 | 44 ± 11 | 45 ± 8 | 47 ± 9 | 40 ±10 |  | 43 ± 6  P=0.868  g=-0.08  CI_95%_=-1.06-0.90 | 44 ± 6  P=0.650  g=-0.22  CI_95%_=--0.76-1.20 | 45 ± 8  P=0.877  g=-0.07  CI_95%_=-1.05-0.91 | 45 ± 6  P=0.943  g=0.03  CI_95%_=-0.95-1.01 | 42 ± 7  P=0.279  g=0.53  CI_95%_=-0.46-1.53 | 44 ± 6  P=0.325  g=-0.48  CI_95%_=-1.48-0.51 |
| **Time delay min** ${\dot{\mathbf{V}}}_{\mathbf{C}\mathbf{O}_{\mathbf{2}}}$ **(s)** | 44 ± 8 | 46 ± 7 | 45 ± 8 | 46 ± 7 | 46 ± 6 | 41 ± 8 |  | 45 ± 5  P=0.880  g=-0.07  CI_95%_=-1.05-0.91 | 44 ± 6  P=0.611  g=-0.25  CI_95%_=-0.74-1.23 | 46 ± 5  P=0.657  g=-0.21  CI_95%_=-1.20-0.77 | 46 ± 5  P=0.902  g=0.06  CI_95%_=-0.92-1.04 | 42 ± 5  P=0.144  g=0.73  CI_95%_=-0.28-1.74 | 44 ± 7  P=0.467  g=-0.35  CI_95%_=--1.34-0.63 |
| **Time delay min HR (s)** | 33 ± 6 | 35 ± 5 | 35 ± 7 | 38 ± 8 | 38 ± 5 | 37 ± 10 |  | 36 ± 6  P=0.391  g=-0.42  CI_95%_=-1.41-0.57 | 36 ± 6  P=0.776  g=-0.14  CI_95%_=-1.12-0.84 | 38 ± 5  P=0.359  g=-0.45  CI_95%_=-1.44-0.54 | 37 ± 4  P=0.691  g=0.19  CI_95%_=-0.79-1.17 | 38 ± 5  P=0.857  g=-0.09  CI_95%_=-1.07-0.89 | 39 ± 6  P=0.634  g=-0.23  CI_95%_=-1.21-0.75 |

**Table S2:** Between groups effect in blood lactate concentration ([La^-^]), rates of perceived exertion (RPE) on a general, muscular and respiratory values of each sinusoid of controls and smokers. Data are shown as mean ± standard deviation (SD).

| **Table 2** | **CONTROLS** | | | | | |  | | **SMOKERS** | | | | | | |  |
| --- | --- | --- | --- | --- | --- | --- | --- | --- | --- | --- | --- | --- | --- | --- | --- | --- |
|  | **Sinusoid 1** | **Sinusoid 2** | **Sinusoid 3** | **Sinusoid 4** | **Sinusoid 5** | **Sinusoid Last** | |  | | **Sinusoid 1** | **Sinusoid 2** | **Sinusoid 3** | **Sinusoid 4** | **Sinusoid 5** | **Sinusoid Last** | |
|  |  |  |  |  |  |  | |  | |  |  |  |  |  |  | |
| **[La^-^] (mM)** | 3.9 ± 0.8 | 3.8 ± 0.8 | 3.8 ± 0.8 | 3.7 ± 0.8 | 3.7 ± 0.8 | 2.6 ± 0.7 | |  | | 3.8 ± 0.9  P=0.934  g=0.08  CI_95%_=-0.90-1.06 | 3.8 ± 0.9  P=0.970  g=-0.10  CI_95%_=-0.88-1.08 | 3.6 ± 1.1  P=0.727  g=0.17  CI_95%_=-0.81-1.15 | 3.6 ± 1.1 P=0.844  g=0.10  CI_95%_=-0.88-1.08 | 3.5 ± 1.2  P=0.705  g=0.19  CI_95%_=-0.79-1.17 | 3.3 ± 1.0  P=0.149  g=-0.74  CI_95%_=-1.75-0.27 | |
| **RPE GENERAL (a.u.)** | 13.1 ± 1.9 | 13.1 ± 1.9 | 13.5 ± 2.1 | 13.8 ± 2.3 | 14.3 ± 2.5 | 19.0 ± 1.1 | |  | | 12.1 ± 1.2  P=0.231  g=0.59  CI_95%_=-0.41-1.59 | 12.9 ± 1.1  P=0.752  g=0.15  CI_95%_=-0.83-1.13 | 13.9 ± 1.6  P=0.688  g=-0.19  CI_95%_=-1.18-0.79 | 14.9 ± 1.1  P=0.237  g=-0.58  CI_95%_=-1.58-0.42 | 15.5 ±1.2  P=0.222  g=-0.60  CI_95%_=-0.37-1.64 | 18.3 ±1.2  P=0.201  g=0.63  CI_95%_=-0.37-1.64 | |
| **RPE MUSCULAR (a.u.)** | 3.9 ± 1.4 | 4.3 ± 1.7 | 5.1 ± 1.9 | 5.1± 1.9 | 5.6 ± 2.0 | 10.0 ± 0.8 | |  | | 3.7 ± 1.4  P=0.723  g=0.17  CI_95%_=-0.81-1.15 | 4.0 ± 1.7  P=0.770  g=0.14  CI_95%_=-0.84-1.12 | 5.4 ± 1.8  P=0.738  g=-0.16  CI_95%_=-1.14-0.82 | 6.3 ± 1.8  P=0.237  g=-0.58  CI_95%_=-1.58-0.42 | 6.8 ± 1.6  P=0.212  g=-0.62  CI_95%_=-1.62-0.39 | 10.0 ± 1.1  P=1.00  g=0.00  CI_95%_=-0.98-0.98 | |
| **RPE RESPIRATORY (a.u.)** | 3.2 ± 1.6 | 3.4 ± 1.7 | 3.9 ± 1.8 | 4.0 ± 1.8 | 4.4 ± 2.1 | 8.0 ± 1.3 | |  | | 2.6 ± 0.9  P=0.353  g=0.45  CI_95%_=-0.54-1.45 | 2.9 ± 0.9  P=0.520  g=-0.31  CI_95%_=-0.67-1.30 | 3.4 ± 0.9  P=0.497  g=0.33  CI_95%_=-0.66-1.32 | 4.6 ± 0.9  P=0.391  g=-0.42  CI_95%_=-0.66-1.32 | 5.0 ± 1.4  P=0.501  g=-0.33  CI_95%_=-1.31-0.66 | 7.3 ± 1.9  P=0.375  g=0.43  CI_95%_=-0.56-1.42 | |

**Table S3:** Within protocols effect in midpoint, amplitude, time delay at maximum and at minimum of the sinusoidal pattern response of pulmonary oxygen uptake ($\dot{V}_{O_{2}}$), ventilation ($\dot{V}_{E}$), carbon dioxide production ($\dot{V}_{\mathrm{CO}_{2}}$) and heart rate (HR) to each sinusoid in controls (table 3a) and smokers (table 3b). Data are shown as mean ± standard deviation (SD). *p<0.05 vs sinusoid 1

| **Table 3a** | **CONTROLS** | | | | | |
| --- | --- | --- | --- | --- | --- | --- |
|  | **Sinusoid 1** | **Sinusoid 2** | **Sinusoid 3** | **Sinusoid 4** | **Sinusoid 5** | **Sinusoid Last** |
|  |  |  |  |  |  |  |
| **Midpoint** ${\dot{\mathbf{V}}}_{\mathbf{O}_{\mathbf{2}}}$ **(ml∙min^-1^)** | 2497 ± 347 | 2523 ± 357  P=1.00  g=-0.07  CI_95%_=-1.05-0.91 | 2536 ± 343  P=0.310  g=-0.11  CI_95%_=-1.09-0.88 | 2573 ± 362  P=0.164  g=-0.20  CI_95%_=-1.18-0.78 | 2578 ± 355  P=0.175  g=-0.22  CI_95%_=-1.20-0.77 | **2710 ± 420***  **P=0.001**  **g=-0.52**  **CI_95%_=-1.52-0.47** |
| **Midpoint** ${\dot{\mathbf{V}}}_{\mathbf{E}}$ **(l∙min^-1^)** | 63 ± 9 | 65 ± 10  P=0.594  g=-0.15  CI_95%_=-1.13-0.83 | 65 ± 10  P=0.102  g=-0.19  CI_95%_=-1.17-0.79 | **67 ± 10***  **P=0.007**  **g=-0.08**  **CI_95%_=-1.06-0.90** | **69 ± 9***  **P<0.001**  **g=-0.11**  **CI_95%_=-1.09-0.87** | **76 ± 12***  **P<0.001**  **g=-0.08**  **CI_95%_=-1.06-0.90** |
| **Midpoint** ${\dot{\mathbf{V}}}_{\mathbf{C}\mathbf{O}_{\mathbf{2}}}$ **(ml∙min^-1^)** | 2374 ± 310 | 2370 ± 338  P=1.00  g=0.01  CI_95%_=-0.97-0.99 | 2382 ± 318  P=1.00  g=-0.02  CI_95%_=-1.00-0.96 | 2411 ± 350  P=1.00  g=-0.11  CI_95%_=-1.09-0.87 | 2410 ± 327  P=1.00  g=-0.11  CI_95%_=-1.09-0.87 | 2431 ± 400  P=1.00  g=-0.15  CI_95%_=-1.13-0.83 |
| **Midpoint HR (b∙min^-1^)** | 145 ± 10 | **148 ± 9***  P=0.077  g=-0.29  CI_95%_=-1.27-0.70 | **150 ± 9***  **P=0.011**  **g=-0.38**  **CI_95%_=-1.37-0.61** | **152 ± 8***  **P=0.002**  **g=-0.52**  **CI_95%_=-1.51-0.48** | **153 ± 8***  **P=0.004**  **g=-0.66**  **CI_95%_=-1.66-0.35** | **165 ± 10***  **P<0.001**  **g=-1.09**  **CI_95%_=-2.14- -0.04** |
|  |  |  |  |  |  |  |
| **Amplitude** ${\dot{\mathbf{V}}}_{\mathbf{O}_{\mathbf{2}}}$ **(ml∙min^-1^)** | 418 ± 44 | 418 ± 59  P=1.00  g=0.00  CI_95%_=-0.98-0.98 | 428 ± 53  P=1.00  g=-0.20  CI_95%_=-1.18-0.79 | 430 ± 62  P=1.00  g=-0.22  CI_95%_=-1.20-0.77 | 436 ± 59  P=1.00  g=-0.33  CI_95%_=-1.31-0.66 | 485 ± 62  P=0.702  g=-1.18  CI_95%_=-2.25—0.12 |
| **Amplitude** ${\dot{\mathbf{V}}}_{\mathbf{E}}$ **(l∙min^-1^)** | 10 ± 3 | 10 ± 2  P=1.00  g=0.01  CI_95%_=-0.98-0.98 | 10 ± 2  P=1.00  g=-0.21  CI_95%_=-1.19-0.78 | 11 ± 2  P=1.00  g=-0.38  CI_95%_=-1.37-0.60 | 11 ± 2  P=0.536  g=-0.44  CI_95%_=-1.43-0.55 | **15 ± 3***  **P=0.003**  **g=-1.62**  **CI_95%_=-2.75- -0.49** |
| **Amplitude** ${\dot{\mathbf{V}}}_{\mathbf{C}\mathbf{O}_{\mathbf{2}}}$ **(ml∙min^-1^)** | 358 ± 53 | 372 ± 53  P=1.00  g=-0.25  CI_95%_=-1.23-0.74 | 377 ± 47  P=1.00  g=-0.35  CI_95%_=1.34-0.64 | 400 ± 42  P=0.702  g=-0.83  CI_95%_=-1.85-0.19 | 401 ± 67  P=1.00  g=-0.66  CI_95%_=-1.66-0.35 | **461 ± 56***  **P=0.048**  **g=-1.78**  **CI_95%_=-2.93- -0.62** |
| **Amplitude HR (b∙min^-1^)** | 10 ± 2 | 11 ± 2  P=1.00  g=-0.26  CI_95%_=-1.24-0.73 | 11 ± 2  P=1.00  g=-0.19  CI_95%_=-1.17-0.79 | 10 ± 2  P=1.00  g=-0.07  CI_95%_=-1.05-0.91 | 10 ± 2  P=1.00  g=-0.11  CI_95%_=-1.09-0.87 | 8 ± 3  P=0.163  g=0.82  CI_95%_=-0.20-1.84 |
|  |  |  |  |  |  |  |
| **Time delay max** ${\dot{\mathbf{V}}}_{\mathbf{O}_{\mathbf{2}}}$ **(s)** | 35 ± 5 | 36 ± 4  P=1.00  g=-0.26  CI_95%_=-1.24-0.73 | 36 ± 7  P=1.00  g=-0.16  CI_95%_=-1.14-0.82 | 35 ± 5  P=1.00  g=0.02  CI_95%_=0.96-1.00 | 35 ± 5  P=1.00  g=-0.07  CI_95%_=-1.05-0.91 | 30 ± 8  P=0.552  g=0.62  CI_95%_=-0.38-1.62 |
| **Time delay max** ${\dot{\mathbf{V}}}_{\mathbf{E}}$ **(s)** | 43 ± 10 | 45 ± 10  P=1.00  g=-0.20  CI_95%_=-1.18-0.78 | 45 ± 12  P=1.00  g=-0.17  CI_95%_=-1.15-0.81 | 45 ± 9  P=1.00  g=-0.21  CI_95%_=-1.19-0.78 | 47 ± 10  P=0.572  g=-0.38  CI_95%_=-1.37-0.61 | 40 ± 9  P=1.00  g=0.28  CI_95%_=-0.70-1.27 |
| **Time delay max** ${\dot{\mathbf{V}}}_{\mathbf{C}\mathbf{O}_{\mathbf{2}}}$ **(s)** | 43 ± 8 | 47 ± 7  P=0.779  g=-0.46  CI_95%_=-1.46-0.53 | 45 ± 8  P=1.00  g=-0.19  CI_95%_=-1.17-0.79 | 46 ± 7  P=1.00  g=-0.36  CI_95%_=-1.35-0.63 | 46 ± 5  P=1.00  g=-0.36  CI_95%_=-1.34-0.63 | 42 ± 8  P=1.00  g=0.22  CI_95%_=-0.76-1.20 |
| **Time delay max HR (s)** | 33 ± 7 | 35 ± 5  P=1.00  g=-0.24  CI_95%_=-1.23-0.74 | 35 ± 7  P=1.00  g=-0.21  CI_95%_=-1.20-0.77 | 38 ± 6  P=0.06  g=-0.70  CI_95%_=-1.71-0.31 | 38 ± 5  P=0.185  g=-0.65  CI_95%_=-1.66-0.36 | 36 ± 7  P=1.00  g=-0.38  CI_95%_=-1.37-0.61 |
|  |  |  |  |  |  |  |
| **Time delay min** ${\dot{\mathbf{V}}}_{\mathbf{O}_{\mathbf{2}}}$ **(s)** | 34 ± 5 | 36 ± 5  P=1.00  g=1.01  CI_95%_=-0.03-2.05 | 34 ± 6  P=1.00  g=0.31  CI_95%_=-0.68-1.29 | 35 ± 5  P=1.00  g=0.48  CI_95%_=-0.52-1.47 | 35 ± 5  P=1.00  g=0.42  CI_95%_=-0.57-1.41 | 31 ± 8  P=1.00  g=0.18  CI_95%_=-0.80-1.17 |
| **Time delay min** ${\dot{\mathbf{V}}}_{\mathbf{E}}$ **(s)** | 42 ± 11 | 46 ± 9  P=0.983  g=-0.18  CI_95%_=-1.16-0.81 | 44 ± 11  P=1.00  g=-0.24  CI_95%_=-1.22-0.75 | 45 ± 8  P=1.00  g=-0.36  CI_95%_=-1.34-0.63 | 47 ± 9  P=0.329  g=0.05  CI_95%_=-0.93-1.03 | 40 ±10  P=1.00  g=-0.21  CI_95%_=-1.19-0.77 |
| **Time delay min** ${\dot{\mathbf{V}}}_{\mathbf{C}\mathbf{O}_{\mathbf{2}}}$ **(s)** | 44 ± 8 | 46 ± 7  P=1.00  g=-  CI_95%_=- | 45 ± 8  P=1.00  g=-  CI_95%_=- | 46 ± 7  P=1.00  g=-  CI_95%_=- | 46 ± 6  P=1.00  g=-  CI_95%_=- | 41 ± 8  P=1.00  g=-  CI_95%_=- |
| **Time delay min HR(s)** | 33 ± 6 | 35 ± 5  P=1.00  g=0.02  CI_95%_=-0.96-1.00 | 35 ± 7  P=1.00  g=-0.27  CI_95%_=-1.25-0.72 | 38 ± 8  P=0.06  g=-0.19  CI_95%_=-1.17-0.80 | 38 ± 5  P=0.169  g=0.50  CI_95%_=-0.50-1.49 | 37 ± 10  P=1.00  g=0.06  CI_95%_=-0.92-1.04 |

| **Table 3b** | **SMOKERS** | | | | | |
| --- | --- | --- | --- | --- | --- | --- |
|  | **Sinusoid 1** | **Sinusoid 2** | **Sinusoid 3** | **Sinusoid 4** | **Sinusoid 5** | **Sinusoid Last** |
|  |  |  |  |  |  |  |
| **Midpoint** ${\dot{\mathbf{V}}}_{\mathbf{O}_{\mathbf{2}}}$ **(ml∙min^-1^)** | 2271 ± 354 | 2281 ± 341  P=1.00  g=-0.03  CI_95%_=-1.01-0.95 | 2303 ± 363  P=0.709  g=-0.08  CI_95%_=-1.06-0.90 | 2315 ± 351  P=1.00  g=-0.12  CI_95%_=-1.10-0.86 | 2324± 342  P=1.00  g=-0.15  CI_95%_=-1.13-0.84 | 2358 ± 388  P=0.487  g=-0.22  CI_95%_=-1.21-0.76 |
| **Midpoint** ${\dot{\mathbf{V}}}_{\mathbf{E}}$ **(l∙min^-1^)** | 60 ± 9 | 60 ± 8  P=1.00  g=-0.01  CI_95%_=-0.99-0.97 | 62 ± 9  P=0.080  g=-0.04  CI_95%_=-1.02-0.94 | **63 ± 8***  **P=0.026**  **g=-0.38**  **CI_95%_=-1.37-0.61** | **64 ± 8***  **P=0.001**  **g=-0.55**  **CI_95%_=-1.54-0.45** | **66 ± 10***  **P=0.049**  **g=-0.67**  **CI_95%_=-1.68-0.34** |
| **Midpoint** ${\dot{\mathbf{V}}}_{\mathbf{C}\mathbf{O}_{\mathbf{2}}}$ **(ml∙min^-1^)** | 2184 ± 350 | 2170 ± 353  P=1.00  g=0.04  CI_95%_=-0.94-1.02 | 2189 ± 372  P=1.00  g=-0.01  CI_95%_=-0.99-0.97 | 2214 ± 370  P=1.00  g=-0.08  CI_95%_=-1.06-0.90 | 2208 ± 355  P=1.00  g=-0.06  CI_95%_=-1.04-0.92 | 2200 ± 380  P=1.00  g=-0.04  CI_95%_=-1.02-0.94 |
| **Midpoint HR (b∙min^-1^)** | 138 ± 16 | 142 ± 16  **P=0.009**  **g=-0.29**  **CI_95%_=-1.27-0.70** | **145 ± 16***  **P<0.001**  **g=-0.43**  **CI_95%_=-1.42-0.56** | **147 ± 16***  **P<0.001**  **g=-0.64**  **CI_95%_=-1.65-0.36** | **149 ± 15***  **P<0.001**  **g=-0.82**  **CI_95%_=-1.85-0.20** | **156 ± 14***  **P<0.001**  **g=-1.80**  **CI_95%_=-2.96- -0.64** |
|  |  |  |  |  |  |  |
| **Amplitude** ${\dot{\mathbf{V}}}_{\mathbf{O}_{\mathbf{2}}}$ **(ml∙min^-1^)** | 411 ± 48 | 405 ± 26  P=1.00  g=0.13  CI_95%_=-0.85-1.11 | 411 ± 42  P=1.00  g=0.00  CI_95%_=-0.98-0.98 | 415 ± 56  P=1.00  g=-0.08  CI_95%_=-1.06-0.90 | 408 ± 56  P=1.00  g=0.05  CI_95%_=-0.93-1.03 | 432 ±104  P=1.00  g=-0.25  CI_95%_=-1.23-0.74 |
| **Amplitude** ${\dot{\mathbf{V}}}_{\mathbf{E}}$ **(l∙min^-1^)** | 10 ± 3 | 9 ± 2  P=1.00  g=0.10  CI_95%_=-0.88-1.08 | 10 ± 3  P=1.00  g=-0.21  CI_95%_=-1.20-0.77 | 11 ± 3  P=1.00  g=-0.40  CI_95%_=-1.39-0.59 | 11 ± 3  P=0.124  g=-0.49  CI_95%_=-1.49-1.50 | 12 ± 5  P=0.126  g=-0.67  CI_95%_=-1.68-0.33 |
| **Amplitude** ${\dot{\mathbf{V}}}_{\mathbf{C}\mathbf{O}_{\mathbf{2}}}$ **(ml∙min^-1^)** | 359 ± 31 | 363 ± 36  P=1.00  g=-0.11  CI_95%_=-1.09-0.87 | 386 ± 47  P=0.932  g=-0.64  CI_95%_=-1.65-0.36 | 381 ± 50  P=1.00  g=-0.51  CI_95%_=-1.51-0.48 | 386 ± 60  P=1.00  g=-0.55  CI_95%_=-1.55-0.45 | 406 ± 91  P=1.00  g=-0.65  CI_95%_=-1.66-0.35 |
| **Amplitude HR (b∙min^-1^)** | 11 ± 4 | 11 ± 3  P=1.00  g=-0.13  CI_95%_=-1.11-0.85 | 11 ± 3  P=1.00  g=-0.10  CI_95%_=-1.08-0.88 | 11 ± 3  P=1.00  g=0.08  CI_95%_=-0.90-1.07 | 11 ± 3  P=1.00  g=0.03  CI_95%_=-0.95-1.01 | 10 ± 3  P=1.00  g=0.20  CI_95%_=-0.78-1.18 |
|  |  |  |  |  |  |  |
| **Time delay max** ${\dot{\mathbf{V}}}_{\mathbf{O}_{\mathbf{2}}}$ **(s)** | 34 ± 8 | 33 ± 7  P=1.00  g=0.11  CI_95%_=-0.87-1.10 | 35 ± 8  P=1.00  g=-0.12  CI_95%_=-1.10-0.86 | 32 ± 8  P=0.837  g=0.27  CI_95%_=-0.71-1.26 | 33 ± 8  P=1.00  g=0.19  CI_95%_=-0.79-1.17 | 34 ± 8  P=1.00  g=0.06  CI_95%_=-0.92-1.04 |
| **Time delay max** ${\dot{\mathbf{V}}}_{\mathbf{E}}$ **(s)** | 43 ± 7 | 44 ± 8  P=1.00  g=-0.06  CI_95%_=-1.04-0.92 | 46 ± 10  P=1.00  g=-0.028  CI_95%_=-1.26-0.71 | 46 ± 8  P=1.00  g=-0.36  CI_95%_=-1.34-0.63 | 41 ± 8  P=1.00  g=0.23  CI_95%_=-0.76-1.21 | 42 ± 7  P=1.00  g=0.08  CI_95%_=-0.90-1.06 |
| **Time delay max** ${\dot{\mathbf{V}}}_{\mathbf{C}\mathbf{O}_{\mathbf{2}}}$ **(s)** | 43 ± 8 | 43 ± 8  P=1.00  g=0.00  CI_95%_=-0.98-0.98 | 43 ± 9  P=1.00  g=0.00  CI_95%_=-0.98-0.98 | 41 ± 8  P=1.00  g=0.23  CI_95%_=-0.75-1.22 | 39 ± 8  P=0.897  g=0.48  CI_95%_=-0.51-1.47 | 44 ± 6  P=1.00  g=-0.08  CI_95%_=-1-06-0.90 |
| **Time delay max HR (s)** | 34 ± 10 | 35 ± 9  P=1.00  g=-0.19  CI_95%_=-1.17-0.79 | 36 ± 10  P=1.00  g=-0.22  CI_95%_=-1.20-0.76 | 33 ± 7  P=1.00  g=0.03  CI_95%_=-0.95-1.01 | 36 ± 8  P=1.00  g=-0.21  CI_95%_=-1.19-0.77 | 37 ± 8  P=1.00  g=-0.38  CI_95%_=-1.37-0.61 |
|  |  |  |  |  |  |  |
| **Time delay min** ${\dot{\mathbf{V}}}_{\mathbf{O}_{\mathbf{2}}}$ **(s)** | 37 ± 6 | 34 ± 5  P=0.852  g=0.51  CI_95%_=-0.49-1.50 | 35 ± 6  P=1.00  g=0.31  CI_95%_=-0.68-1.29 | 34 ± 6  P=1.00  g=0.48  CI_95%_=-0.52-1.47 | 34 ± 6  P=1.00  g=0.42  CI_95%_=-0.57-1.41 | 36 ± 5  P=1.00  g=0.18  CI_95%_=-0.80-1.17 |
| **Time delay min** ${\dot{\mathbf{V}}}_{\mathbf{E}}$ **(s)** | 43 ± 6 | 44 ± 6  P=1.00  g=-0.18  CI_95%_=-1.16-0.81 | 45 ± 8  P=1.00  g=-0.34  CI_95%_=-1.22-0.75 | 45 ± 6  P=1.00  g=-0.36  CI_95%_=-1.34-0.63 | 42 ± 7  P=1.00  g=0.05  CI_95%_=-0.93-1.03 | 44 ± 6  P=1.00  g=-0.21  CI_95%_=-1.19-0.77 |
| **Time delay min** ${\dot{\mathbf{V}}}_{\mathbf{C}\mathbf{O}_{\mathbf{2}}}$ **(s)** | 45 ± 5 | 44 ± 6  P=1.00  g=0.02  CI_95%_=-0.96-1.00 | 46 ± 5  P=1.00  g=-0.27  CI_95%_=-1.25-0,72 | 46 ± 5  P=1.00  g=-0.19  CI_95%_=-1.17-0.80 | 42 ± 5  P=0.777  g=0.50  CI_95%_=-0.50-1.49 | 44 ± 7  P=1.00  g=0.06  CI_95%_=-0.92-1.04 |
| **Time delay min HR (s)** | 36 ± 6 | 36 ± 6  P=1.00  g=-0.04  CI_95%_=-1.02-0.94 | 38 ± 5  P=1.00  g=-0.40  CI_95%_=-1.39-0.59 | 37 ± 4  P=1.00  g=-0.23  CI_95%_=-1.21-0.75 | 38 ± 5  P=1.00  g=-0.39  CI_95%_=-1.38-0.60 | 39 ± 6  P=1.00  g=-0.50  CI_95%_=-1.49-0.50 |

**Table S4:** Within protocols effect in blood lactate concentration ([La^-^]), rates of perceived exertion (RPE) on a general, muscular and values of each sinusoid of controls (table 4a) and smokers (table 4b). Data are shown as mean ± standard deviation (SD). *p<0.05 vs sinusoid 1.

| **Table 4a** | **CONTROLS** | | | | | |
| --- | --- | --- | --- | --- | --- | --- |
|  | **Sinusoid 1** | **Sinusoid 2** | **Sinusoid 3** | **Sinusoid 4** | **Sinusoid 5** | **Sinusoid Last** |
|  |  |  |  |  |  |  |
| **[La^-^] (mM)** | 3.9 ± 0.8 | 3.8 ± 0.8  P=1.00  g=0.03  CI_95%_=-0.95-1.01 | 3.8 ± 0.8  P=1.00  g=0.11  CI_95%_=-0.88-1.09 | 3.7 ± 0.8  P=1.00  g=0.22  CI_95%_=-0.77-1.20 | 3.7 ± 0.8  P=1.00  g=0.20  CI_95%_=-0.78-1.18 | **2.6 ± 0.7***  **P=0.001**  **g=1.65**  **CI_95%_=0.52-2.79** |
| **RPE GENERAL (a.u..)** | 13.1 ± 1.9 | 13.1 ± 1.9  P=1.00  g=0.00  CI_95%_=-0.98-0.98 | 13.5 ± 2.1  P=1.00  g=-0.18  CI_95%_=-1.16-0.80 | 13.8 ± 2.3  P=1.00  g=-0.28  CI_95%_=-1.26-0.70 | 14.3 ± 2.5  P=0.235  g=-0.48  CI_95%_=-1.48-0.51 | **19.0 ± 1.1***  **P<0.001**  **g=-3.62**  **CI_95%_=-5.22- -2.03** |
| **RPE MUSCULAR (a.u.)** | 3.9 ± 1.4 | 4.3 ± 1.7  P=1.00  g=-0.19  CI_95%_=-1.18-0.79 | **5.1± 1.9***  **P=0.012**  **g=-0.64**  **CI_95%_=-1.65-0.36** | **5.1± 1.9***  **P=0.027**  **g=-0.68**  **CI_95%_=-1.69-0.33** | **5.6 ± 2.0***  **P=0.001**  **g=-0.89**  **CI_95%_=-1.91-0.14** | **10.0 ± 0.8***  **P<0.001**  **g=-5.17**  **CI_95%_=-7.21- -3.13** |
| **RPE RESPIRATORY (a.u.)** | 3.2 ± 1.6 | 3.4 ± 1.7  P=1.00  g=-0.12  CI_95%_=-1.10-0.86 | **3.9 ± 1.8***  **P=0.006**  **g=-0.86**  **CI_95%_=-1.89-0.16** | **4.0 ± 1.8***  **P=0.031**  **g=-2.19**  **CI_95%_=-3.43- -0.95** | **4.4 ± 2.1***  **P=0.102**  **g=-1.97**  **CI_95%_=-3.16- -0.77** | **8.0 ± 1.3***  **P<0.001**  **g=-2.99**  **CI_95%_=-4.42- -1.57** |

| **Table 4b** | **SMOKERS** | | | | | |
| --- | --- | --- | --- | --- | --- | --- |
|  | **Sinusoid 1** | **Sinusoid 2** | **Sinusoid 3** | **Sinusoid 4** | **Sinusoid 5** | **Sinusoid Last** |
|  |  |  |  |  |  |  |
| **[La^-^] (mM)** | 3.8 ± 0.9 | 3.8 ± 0.9  P=1.00  g=0.04  CI_95%_=-0.94-1.02 | 3.6 ± 1.1  P=0.669  g=0.17  CI_95%_=-0.81-1.16 | 3.6 ± 1.1  P=1.00  g=0.19  CI_95%_=-0.79-1.18 | 3.5 ± 1.2  P=1.00  g=0.27  CI_95%_=-0.72-1.25 | 3.3 ± 1.0  P=0.354  g=0.51  CI_95%_=-0.48-1.51 |
| **RPE GENERAL (a.u..)** | 12.1 ± 1.2 | **12.9 ± 1.1***  **P<0.001**  **g=-0.60**  **CI_95%_=-1.60-0.40** | **13.9 ± 1.6***  **P<0.001**  **g=-1.18**  **CI_95%_=-2.24- -0.11** | **14.9 ± 1.1***  **P<0.001**  **g=-2.19**  **CI_95%_=-3.43- -0.95** | **15.5 ±1.2***  **P<0.001**  **g=-2.61**  **CI_95%_=-3.95- -1.28** | **18.3 ±1.2***  **P<0.001**  **g=-4.80**  **CI_95%_=-6.73- -2.87** |
| **RPE MUSCULAR (a.u.)** | 3.7 ± 1.4 | 4.0 ± 1.7  P=1.00  g=-0.19  CI_95%_=-1.17-0.79 | **5.4 ± 1.8***  **P<0.001**  **g=-1.00**  **CI_95%_=-2.04-0.04** | **6.3 ± 1.8***  **P<0.001**  **g=-1.53**  **CI_95%_=-2.65- -0.42** | **6.8 ± 1.6***  **P<0.001**  **g=-1.95**  **CI_95%_=-3.14- -0.76** | **10.0 ± 1.1***  **P<0.001**  **g=-4.82**  **CI_95%_=-6.75- -2.88** |
| **RPE RESPIRATORY (a.u.)** | 2.6 ± 0.9 | 2.9 ± 0.9  P=0.490  g=-0.39  CI_95%_=-1.38-0.60 | **3.4 ± 0.9***  **P=0.001**  **g=-0.38**  **CI_95%_=-1.37-0.61** | **4.6 ± 0.9***  **P<0.001**  **g=-0.45**  **CI_95%_=-1.44-0.54** | **5.0 ± 1.4***  **P<0.001**  **g=-0.59**  **CI_95%_=-1.59-0.41** | **7.3 ± 1.9***  **P<0.001**  **g=-3.08**  **CI_95%_=-4.53- -1.63** |
